# Survey of faculty development in four Israeli medical schools: clinical faculty development is inadequate and clinical teaching is undervalued in Israeli faculties of medicine

**DOI:** 10.1186/s13584-021-00438-0

**Published:** 2021-02-08

**Authors:** Simon Nothman, Michael Kaffman, Rachel Nave, Moshe Y. Flugelman

**Affiliations:** 1 Department of Education, Ruth and Bruce Faculty of Medicine, Technion IIT, Haifa, Israel; 2 Department of Obstetrics and Gynaecology, Haemek Medical Center, Afula, Israel; 3 Department of Family Practice, Clalit Health Services, Haifa and Western Galilee District, Haifa, Israel; 4 Department of Cardiovascular Medicine, Lady Davis Carmel Medical Center, 7 Michal St., Haifa, Israel

**Keywords:** Medical education, Faculty development, Clinical teaching, Health system planning

## Abstract

**Background:**

Teaching medical students is a central part of being a doctor, and is essential for the training of the next generation of physicians and for maintaining the quality of medicine. Our research reviews the training that physicians in Israel receive as teachers of clinical clerkships, and their thoughts regarding teaching students. The importance of faculty development cannot be overstated, for securing quality medicine and physician empowerment.

**Methods:**

This study was based on a survey conducted among physicians teaching at Israeli medical schools. The survey was conducted using an online questionnaire sent to clinical teachers according to lists received from the teaching units of the faculties, department heads, and other clinical teachers. Participation in the study was anonymous.

**Findings:**

Of 433 invited physicians, 245 (56%) from three departments (internal medicine, paediatrics, obstetrics and gynaecology) of four faculties of medicine in Israel, out of five total, completed the questionnaire. Only 35% of the physicians reported having received training for their role as teachers, most of these participated in a short course of up to 2 days. There were significant differences between the Technion and the other schools. Technion teachers without academic appointment had higher rates of pedagogic training. The same was true in regard to Technion teachers, either residents or young specialist. Significant gaps were reported between the content covered in the training and the topics the doctors felt they would want to learn. The clinicians who participated in the survey expressed that clinical teaching was less valued and more poorly remunerated than research, and that improved compensation and perceived appreciation would likely improve the quality of clinical teaching.

**Conclusions:**

Of the one-third of the physicians surveyed who had received some training in clinical teaching, the training was perceived as inadequate and not aligned with their needs. There was a significant difference in rates of pedagogic training between the Technion and other medical schools. In addition, most clinical teachers surveyed felt that teaching students is inadequately valued. Due to its focus on just three disciplines, and higher relative number participants from the Technion faculty of medicine, our survey may not fully represent the activities of the faculties of medicine in Israel. Nevertheless, given the importance of clinical teaching of medical students, our findings argue for increasing faculty development and educational training of physicians in clinical settings, for recognizing the importance of teaching in academic and professional promotion processes.

**Supplementary Information:**

The online version contains supplementary material available at 10.1186/s13584-021-00438-0.

## Introduction

Beyond the role of clinician, a significant and integral part of a doctor’s profession is to teach, and to educate the next generation of doctors. The term “doctor” derives from the Latin word to teach, and the importance of teaching in medicine is featured even in the Hippocratic oath [1].

High quality teaching is recognized as a precondition for quality medicine. Good clinical teachers [2] and the presence of students in clinical settings [3] raise the level of clinical care. Physicians who teach tend to report improvements in their own knowledge; and teaching may also help prevent burnout [4, 5], define their professional role, and empower physicians [6, 7].

Comprehensive training of physicians as teachers, with an emphasis on professionalism and clinical teaching methods, is essential to faculty development programs [8, 9]. The Edinburgh Declaration, published at the 1988 World Conference on Medical Education states, among other conclusions, a requirement to “train teachers as educators, not content experts alone, and to reward excellence in this field as fully as excellence in biomedical research or clinical practice” [10]. Likewise, the British General Medical Council (GMC) has declared that doctors engaging in teaching must acquire the skills, attitudes, and practices of a competent teacher [10]. The GMC has further stated that medical schools must ensure that everyone involved in educating students has the requisite knowledge and skills, and that doctors should be provided with appropriate training to teach students [2].

Despite the centrality of education in medicine, most teaching physicians do not receive sufficient training [11, 12] and many receive no training at all in education and teaching [13]. Thus, inadequate pedagogic training is a universal problem that endangers future generations of students and physicians alike. In 2014, an international committee performed an external review of the medical schools in Israel, and expressed concerns regarding the training and support of the educators in medical schools and recommended higher recognition and compensation for teaching [14, 15]. For the full committee report see Supplementary data, the section related to faculty development is in pages 19, 20, and 46.

Various models have been developed for improving teaching skills. These range from comprehensive programs over months or years, to short training courses of 1–3 days [16].These interventions seem to improve the quality of teaching, clinical teachers’ self-assessment of their educational abilities [16, 17] and their evaluation by senior colleagues [18]. Post-implementation evaluations showed such programs to be feasible [19], and relatively inexpensive [20]. Moreover, they demonstrated a positive effect on knowledge and the quality of teaching [19]. Long-term effects [21] include increased desire to continue teaching in the future [22].

Medical education in Israel has been evolving significantly in recent years [23–25], but there is a lack of information regarding the training of teaching physicians. Needs assessment is essential for the establishment of faculty development programs [26, 27]. Lack of these types of information limits the development of suitable programs for improving clinical teaching. Moreover, there is no regulation of faculty development programs in Israel such as by the joint forum of the deans of faculties of medicine.

In this context, we conducted a research project whose main purpose was to evaluate and characterize the perceptions of clinical teachers of medical students about the training they received in clinical teaching. The findings of our study should be used in planning the future of medical education in Israel.

## Methods

This study consisted of an online survey of physicians who are responsible for teaching medical students in in-hospital clinical clerkships in internal medicine, paediatrics, and obstetrics and gynaecology. Participants were identified based on recommendations from medical faculties’ clinical education units, heads of clinical departments and clinician educator colleagues. The survey aimed to assess the training the participants received to perform their roles as medical teachers, their perceptions of this training, and their attitudes toward teaching. The questionnaire included demographic information, 87 closed questions (Likert scale, scored from 1 to 5), and one open question. The data were analysed according to sub-populations, such as medical specialty, holders of an official academic appointment, and quantity of clinical and teaching experience. We focused on three disciplines and in-hospital teaching, as most teaching in Israel is still conducted in hospitals.

The questionnaire was developed by the authors of this manuscript and is based on their experience in teaching, managing faculty educational systems, and running faculty development programs. The format is adapted from that of Foster et al. [17], as suitable to the ecosystem in Israel. From lists of names provided by the relevant medical schools, we invited 433 physicians to participate in the survey. Of them, 253 completed the questionnaire (58.4% response rate). We approached potential participants up to 3 times. Of the responders, 40% replied on the first time and the rest responded equally after the second and third reminders.

Participation in the survey was anonymous and was preceded by each participant’s “signing” an online informed consent form. The questionnaire was built using Jotform (www.jotform.com) and quantitative data processing was performed in SPSS v21 and Microsoft Excel 15.24. Tests of statistical significance included χ^2^, the one-sided t-test, and one-sided ANOVA as appropriate.

We tested the differences between the Technion medical school and the other 3 medical schools grouped together (Tel Aviv University, Hebrew University, Jerusalem, and Ben Gurion university) comparing rates of pedagogic training, content of training and the length.

The study was planned as a survey and thus no primary outcome was defined for the purpose of calculating the number of participants required. The survey was open to respondents between June and December 2017.

The study was carried out with the approval of the Ethics Committee for Social and Behavioural Sciences of the Technion, Israel Institute of Technology.

## Results

### Characteristics of the respondents to the survey

Of the 433 physicians invited to participate in the survey 253 (58.4%) completed the questionnaire. The rate of participation in the survey out of those approached from the Technion faculty of medicine was 104/152 (68.4%) and from the other universities 149/281 (53%)(*p* < 0.002). Eight doctors from Bar Ilan University Medical Faculty completed the questionnaire without knowing that their faculty refused to participate, and were subsequently removed from the analysis. Table 1 presents demographic data and the experience of the physicians who participated in the survey. Of the 245 participants included in the statistical analysis, 120 (49%) were women, 123 (50%) were internal medicine specialists, and only 76 (31%) held an academic appointment. Forty-two percent of the participants were from the Technion Medical School; and smaller proportions were from the other three faculties.

**Table 1:** Demographic data and experience of the physicians who participated in the survey

Population	Number of participants	Percentage of all participants
**Gender**		
Male	120	49.0
Female	125	51.0
**Medical specialty**		
Internal medicine	123	50.2
Obstetrics & gynaecology	45	18.4
Paediatrics	77	31.4
**Medical school**		
Ben Gurion University of the Negev	28	11.4
Hebrew University of Jerusalem	29	11.8
Technion Israel Institute of Technology, Haifa	104	42.4
Tel Aviv University	84	34.3
**Years of professional experience (excluding internship)**		
0–2 years	25	10.2
3–5 years	59	24.1
6–10 years	102	41.6
Over 10 years	59	24.1
**Years of educational experience**		
0–2 years	93	38.0
3–5 years	46	18.8
6–10 years	67	27.3
Over 10 years	39	15.9
**Holds an official academic appointment**		
Yes	76	31.0
No	169	69.0
**Stage of professional development when completing the survey**		
Junior Resident (prior to Part 1 exam)	30	12.2
Senior Resident (following Part 1 exam)	73	29.8
Junior Specialist (less than 5 years’ experience as a specialist)	82	33.5
Senior Specialist (more than 5 years’ experience as a specialist)	60	24.5
**Stage of professional development when first filling the role of clinical tutor**		
Junior Resident (prior to Part 1 exam)	124	50.6
Senior Resident (following Part 1 exam)	88	35.9
Junior Specialist (less than 5 years’ experience as a specialist)	18	7.3
Senior Specialist (more than 5 years’ experience as a specialist)	15	6.1

### Pedagogic training received by the respondents

Of the 245 physicians in the cohort, only 86 (35%) reported receiving formal training as a teacher (Table 2). No significant differences were found between those who did and did not receive formal training, according to medical speciality, stage of professional development or academic appointment (Table 2). More than half the participants from Technion Medical School reported having received training; the proportions from the other medical schools was smaller. However, the higher proportion of participants from the Technion than from the other faculties raises the possibility of a selection bias.

**Table 2:** Rates of undertaking training for a clinical teaching role

Population	Undertook training	Did not undertake training	Training rate (%)	***p*** value
All participants (*n* = 245)	86	159	35.1	.000
Other Medical Schools (*n* = 141)	33	108	23.4%	
Technion Medical School (*n* = 104)	53	51	51.0%	
**By specialty:**				
Internal medicine (*n* = 45)	13	32	28.9	.003
Other Medical Schools (*n* = 23)	2	21	8.7%	
Technion Medical School (*n* = 22)	11	11	50.0%	
Obstetrics & Gynecology (*n* = 123)	48	75	39.0	.042
Other Medical Schools (*n* = 67)	21	46	31.3%	
Technion Medical School (*n* = 56)	27	29	48.2%	
Pediatrics (*n* = 77)	25	52	32.5	.001
Other Medical Schools (*n* = 51)	10	41	19.6%	
Technion Medical School (*n* = 26)	15	11	57.7%	
**By academic appointment**				
Holds an academic appointment (*n* = 76)	22	54	28.9	ns
Other Medical Schools (*n* = 54)	11	43	20.4%	
Technion Medical School (*n* = 22)	11	11	50.0%	
Does not hold an academic appointment (*n* = 169)	64	105	37.9	.000
Other Medical Schools (*n* = 87)	22	65	25.3%	
Technion Medical School (*n* = 82)	42	40	51.2%	
**By stage of professional development when completing the survey**				
Senior Specialist (more than 5 years’ experience as a specialist) (*n* = 30)	13	17	43.3	.015
Other Medical Schools (*n* = 38)	6	32	15.8%	
Technion Medical School (*n* = 22)	10	12	45.5%	
Junior Specialist (less than 5 years’ experience as a specialist) (*n* = 73)	31	42	42.5	.007
Other Medical Schools (*n* = 46)	9	37	19.6%	
Technion Medical School (*n* = 36)	17	19	47.2%	
Senior Resident (following Part 1 exam) (*n* = 82)	26	56	31.7	ns
Other Medical Schools (*n* = 38)	14	24	36.8%	
Technion Medical School (*n* = 35)	17	18	48.6%	
Junior Resident (prior to Part 1 exam) (*n* = 60)	16	44	26.7	.002
Other Medical Schools (*n* = 19)	4	15	21.1%	
Technion Medical School (*n* = 11)	9	2	81.8%	
**By stage of professional development when first performing the role of clinical tutor**				
Senior Specialist (more than 5 years’ experience as a specialist) (*n* = 124)	50	74	40.3	ns
Other Medical Schools (*n* = 10)	1	9	10.0%	
Technion Medical School (*n* = 5)	2	3	40.0%	
Junior Specialist (less than 5 years’ experience as a specialist) (*n* = 88)	27	61	30.7	ns
Other Medical Schools (*n* = 10)	2	8	20.0%	
Technion Medical School (*n* = 8)	4	4	50.0%	
Senior Resident (following Part 1 exam) (*n* = 18)	6	12	33.3	ns
Other Medical Schools (*n* = 47)	11	36	23.4%	
Technion Medical School (*n* = 41)	16	25	39.0%	
Junior Resident (prior to Part 1 exam) (*n* = 15)	3	12	20.0	.000
Other Medical Schools (*n* = 74)	19	55	25.7%	
Technion Medical School (*n* = 50)	31	19	62.0%	

Table 3 presents characteristics of the pedagogic training physicians received, and its timing during their medical training and career development. Of those who received training, 47 (55%) received training of 1–2 days’ duration. For 57 (66%), the training was provided by both education professionals and practicing physicians (Table 3).

**Table 3:** Duration, methods, and presenters of training in medical education

Variable	Number (n)	Percent of those who undertook pedagogic training (*n* = 86)	Percent of all participa-nts (*n* = 245)	Percent of those who undertook pedagogic training			Percent of all participants		
				Technion School of Medicine (*N* = 53)	Other schools (*N* = 33)	*p* value	Technion Med School (*N* = 104)	Other schools (*N* = 141)	*p* value
Duration of training									
Less than one day	20	23.3	8.2	28.3	15.2	.000	15.4	3.5	.000
1–2 days	47	54.7	19.2	39.6	78.8		20.2	18.4	
3–7 days	9	10.5	3.7	13.2	6.1		6.7	1.4	
Over a week	10	11.6	4.1	18.9	0.0		9.6	0.0	
Teaching method									
Lectures	9	10.5	3.7	11.3	9.1	ns	5.8	2.1	.000
Workshops	33	38.4	13.5	54.7	45.5		17.3	10.6	
Mixed method	44	51.2	18.0	34.0	45.5		27.9	10.6	
Training led by:									
Education professionals	17	19.8	6.9	30.2	3.0	.000	15.4	0.7	.000
Medical practitioners	12	14.0	4.9	5.7	27.3		2.9	6.4	
Combination of education and medical	57	66.3	23.3	64.2	69.7		32.7	16.3	
Undertook pedagogic training prior to acting in a clinical tutor role:									
As a student	2	2.3	0.8	0.0	6.1	.023	0.0	1.4	.000
As a resident	59	68.6	24.1	62.3	75.8		31.7	17.7	
As a specialist	11	12.8	4.5	11.3	15.2		5.8	3.5	
Total	72	83.5	29.4						

### Attitudes of the respondents to their pedagogic training and to teaching

The extent that various aspects of medical education were taught was compared to the extent that the participants would have wanted them to be taught (Table 4). According to the responses, all the topics examined were taught to a markedly lesser extent than the participants of the survey would have desired. While a significant disparity was found for all the topic areas, some aspects of medical education showed particularly large gaps between the actual and desired situations (Table 4). These topics included: preparing examinations and learning materials, lesson planning, discussing clinical and ethical challenges, and principles of bedside teaching. The findings attest to the need to train clinical teachers in these content areas (Table 4). This also emphasizes the importance of needs assessment before designing a faculty development program, as noted previously [19, 20].

**Table 4:**
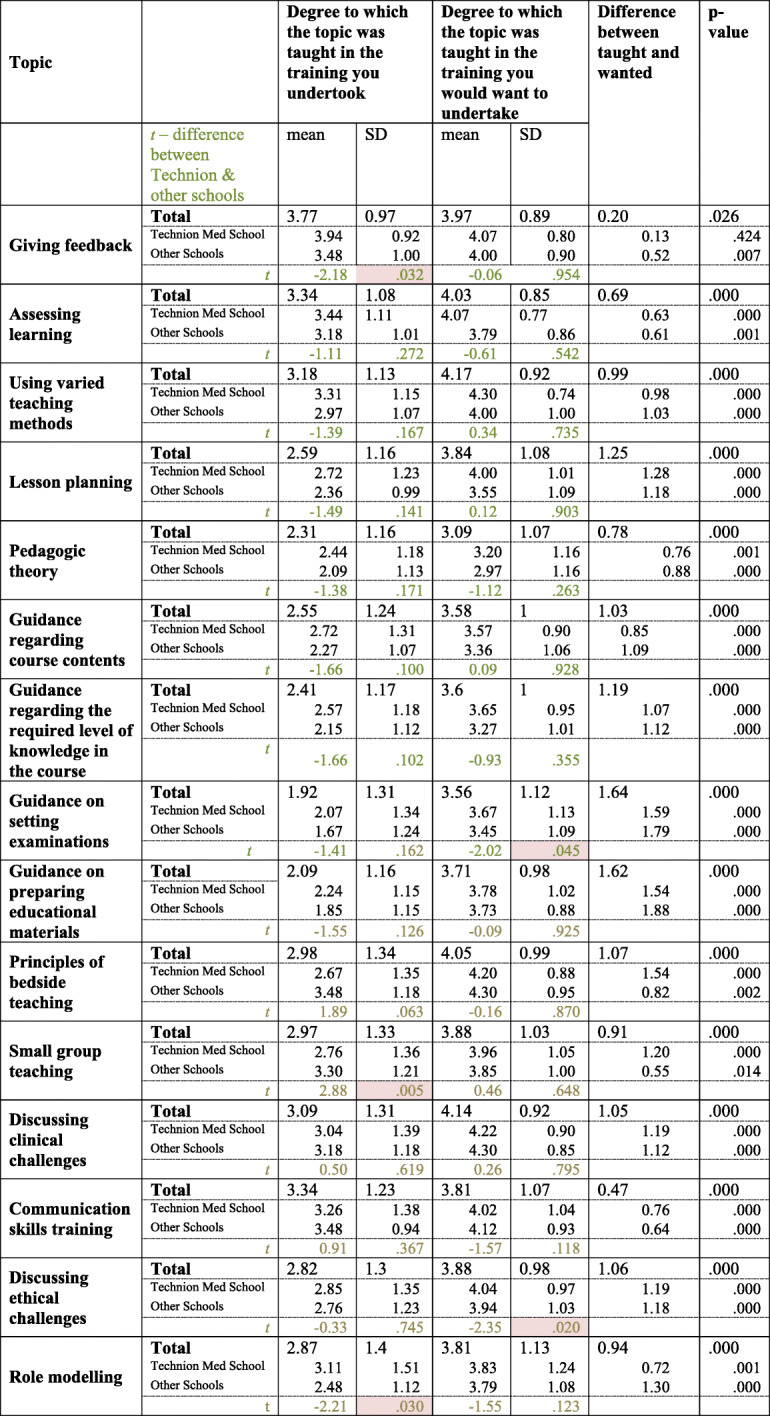
Comparison between the degree to which topics were reported to have been taught in pedagogic training, and the degree to which they would be taught in a course the participants would want to undertake. (“Mark the most appropriate option regarding the degree to which the topic was taught in the training you undertook / would want to undertake”). Scale: 1 – not at all, 5 – to a very great degree

Technion Med School *n* = 54, Other schools = 33, Shaded cells = significant difference between the Technion and the other schools

In regard to content of training, there were differences in responses between the Technion and the other schools amongst those who had undertaken educational training as reflected in the replies in the survey (*n* = 53 for the Technion and *n* = 33 for the pooled respondents from the other three schools). In terms of the content of the training received, respondents reported more of a focus on Giving Feedback at the Technion than other schools (3.94 vs 3.48/5, *p* = 0.032), and Role Modeling (3.11 vs 2.48/5, *p* = 0.03), and less emphasis on teaching about Small Group Teaching (2.76 vs 3.30/5, *p* = 0.005). Statistically significant differences between the Technion and the other schools were not found regarding other topics of study.

The significant difference between clinical teachers’ desires of course content and its reported content was found consistently at both the Technion and the other schools, across all topics, excepting Giving Feedback for which no difference was found at the Technion (Table 5).

**Table 5:** differences between the medical schools in Israel

	Technion *n* = 104	Other medical schools *n* = 141	*p* value	Whole group *n* = 245
Survey participants per discipline (%):				
Internal medicine	22(21%)	23(16%)	0.003	45(29%)
Pediatrics	26(25%)	51(37%)	0.042	77(39%)
Gynecology	56(54%)	67(47%)	0.001	123(32%)
Physicians in training	46 (44%)	57 (40%)	ns	103 (42%)
Underwent educational training (%)	53 (51%)	33 (23%)	0.000	86 (35%)
Academic appointments	22(21%)	54(38%)	0.004	76(31%)
Length of training (% of those who undertook training):	*N* = 53	*N* = 33		*N* = 86
Less than one day	15 (28%)	5 (15%)	0.000	20 (23%)
1–2 days	21 (40%)	26 (79%)		47 (55%)
3–7 days	7 (13%)	2 (6%)		9 (10%)
Over a week	10 (19%)	0 (0%)		10 (12%)

The respondents to the survey indicated a very high level of motivation to engage in teaching students (Supplementary Table 1). The leading factors mentioned as important in increasing the motivation to teach are the understanding that teaching improves the tutor’s learning, the tutor’s desire to contribute to the next generation of physicians, and the understanding that teaching is integral to a physician’s role. Financial rewards, a desire to please superiors, and requirements for academic appointment received lower scores. Respondents who had received training in clinical instruction felt that the training moderately improved their motivation to teach (Supplementary Table 1).

Participants indicated a relatively high level of self-confidence in most domains of medical education, with no significant difference between sub-populations. They also stated moderate improvement in their self-confidence as a result of educational training (Supplementary Table 2).

Survey participants rated several personal attributes as important in a clinical teacher. No significant difference were found between the different attributes (Supplementary Table 3).

The comparison between the Technion and the other three schools showed significant differences between rates of pedagogic training rates and length as shown in Tables 3 and 4. There were also significant differences in content of training but also there were similarities in training deficiencies identified by physicians in the 4 medical schools.

### Perceptions of the respondents of the importance their faculty attributes to teaching

As a whole, respondents indicated that teaching is inadequately acknowledged and recognized, and felt teaching to be more poorly recognized and remunerated than research (Supplementary Table 4). These findings were consistent across all sub-populations assessed (Supplementary Tables 5–7), with the exception of one question in which a statistically significant difference between respondents of different medical schools was found (Supplementary Table 5).

Participants were asked to respond to the open-ended question, “What in your opinion are the most important aspects of the training of physicians for teaching in the clinical environment?” (Supplementary Table 8). Three broad themes were identified in the responses: 1. systemic factors, 2. reward and recognition, 3. faculty development plans with clear goals and objectives.

Systemic factors and the need to establish formal teaching training were considered important particularly by veteran teachers. Less experienced teachers commented more about the desired content in the training course. Moreover, experienced teachers desired assistance to serve as role models and to teach in an inspiring manner, while younger teachers had more basic requirements: guidance on clerkship requirements, lesson planning and teaching methods.

## Discussion

Several major findings emerged from this survey. First, only 35% of clinical teachers in medical clerkships of internal medicine, paediatrics, and obstetrics and gynaecology in Israeli medical schools reported having received specific training for medical education. Of those, about one-fifth received such training only after beginning their roles as tutors. Thus, under 30% of medical students’ clinical teachers had received training prior to undertaking this important role. This rate is very low, considering the WHO recommendation [28] and the requirement in countries such as the UK [10], the Netherlands [29], and Germany [30] that anyone involved in educating medical students first undertake training for that role.

This low rate of educational training did not differ between the specialties surveyed, nor between levels of seniority or according to having an official academic appointment. Notably, a statistically significant difference in the training rate was found between medical schools. This likely reflects a low rate of participation in the survey in some schools, and not a true difference. Even in the medical school with the highest rate of educational training, the rate was only 50% – well below the European standards. This indicates that all faculties in Israel must substantially improve the training of teaching physicians. Medical education in Israel is a dynamic field that has evolved substantially in recent years [15, 23–25]. Opportunities for advanced studies in medical education are available in most universities that teach medicine in Israel, and academic promotion based on teaching is established in most faculties. These platforms are in line with the international committee recommendations [14].

The need to train physicians as teachers includes the need for in-depth programs of faculty development; these are essential for improving the quality of teaching and evaluation [8, 26, 31]. A policy requiring formal pedagogical training would help ensure that physicians who teach students have the requisite knowledge, attitudes and skills, and meet international standards [31–33]. The importance of faculty development programs is highlighted by the empowerment physicians gain when they are trained to perform functions outside their roles of physicians; for example, as managers [7] and teachers [6]. Apparently, such programs help physicians identify with their roles as teachers and managers, and contribute to their career choices.

Of the respondents to our survey who received teaching training, most undertook a short course of less than 2 days (Table 3). While short courses were once the standard [11] for training medical educators, longitudinal training over the course of a teacher’s career is preferred. Studies have shown positive effects of this approach in medical education [31, 34, 35].

The results of the survey also demonstrated gaps in the content of the training that clinician-teachers receive. The respondents indicated that the topics most studied in pedagogic training of Israeli doctors are the provision of feedback, evaluating learning, and training for interpersonal communication skills. The vocabulary that medical educators use is many times enigmatic to practicing physicians. With exposure to faculty development program, terms such as “professionalism” become a part of the vocabulary of physicians, and the use of such terms is subsequently transferred to students. Conversely, the topics that the respondents most desired instruction were the use of diverse teaching methods, the discussion of clinical challenges and principles of bedside teaching. Further, of all the topics addressed in the survey, a significant difference was found between the extent to which teachers desired the subjects be taught, and the extent to which they were reportedly taught.

Generally, faculty development programs are based on theoretical frameworks, and the experience and opinions of experts. While this content may be suitable for experts and researchers in medical education, it does not necessarily suit the needs of doctors who are primarily clinicians and who teach students as a secondary role [29]. Teaching theory must also be tailored to the relevant audience and environment, and this is a realm for experienced educators.

Our findings corroborate previous studies that found that doctors desire to improve in teaching methods and strategies [36] and are interested in training in small group work, basic teaching skills and assessment of students [11]. For these reasons, assessing the needs of participants in pedagogic training, as carried out in this survey, is of critical importance [27], and should serve as a basis for faculty development programs [26].

As noted earlier, internal factors (e.g. the desire to contribute, the recognition of the importance of teaching in a physician’s role) were rated as having more influence in increasing motivation to teach than were external factors (remuneration, academic titles). This concurs with previous studies [4, 37, 38]. This said, the standard deviations of the external factors were relatively broad, indicating that these are nonetheless important to many clinician-educators. Similarly, previous reports showed that inadequate reward for teaching reduces the motivation to teach [39]. These data are important for optimising recruitment, development, and the retention of academic faculty in clinical teaching [38].

The reported improvement in motivation following pedagogic training is an additional reason for medical schools to provide pedagogic training to their teaching staff. This suggests that tailoring training programs to the needs of clinician-teachers may further increase their motivation.

As we observed, regardless of faculty development programs, self-confidence may be short-lived. This is because lack of investment in training and personal and professional development usually leads to burnout and loss of empathy and self-confidence [7].

Participants’ perception that medical education is poorly recognized and remunerated is an important finding. Notably, such opinions did not vary by specialty, by educational or professional experience, or by academic appointment. Differences were found between medical schools in attitudes to the statement: “I believe that good teaching is appropriately recognized and rewarded”. However, we suppose that this is not a true finding, but rather represents sampling bias due to the low number of participants from two of the medical schools. The sentiments expressed by the survey participants seem to reflect a situation that has been previously described [40]. Specifically, research tends to be more valued than teaching. The former offers easily evaluated outcome measures such as the publication of articles and receipt of grants, and a tangible impact on remuneration [41] and academic promotion [42]. In principle, academic promotion committees recognize the importance of teaching [43]. However, in practice teaching does not seem to constitute a major consideration in promotion decisions [44].

Various methods have been described for encouraging and improving medical instruction. These include the establishment of academic advancement tracks on the basis of teaching and educational activity [37], the development of departmental vision and strategy to promote teaching, and the establishment of specific committees for the academic promotion of clinical teachers [45]. The Association of American Medical Colleges has developed a “toolbox” for the objective assessment of academic educational activity, and for enabling consistent ranking of teachers for academic advancement [41]. Teachers in medical schools in Israel are expected to teach in addition to performing their professional duties as physicians. Lack of allocated and regulated time for teaching is probably a main topic that must be changed to improve the way teaching is recognized and appraised.

Responses to the open question “What are the aspects of greatest importance in your opinion regarding the training of doctors for teaching in the clinical environment?” generally reflect responses to the Likert-scale questions, and strengthen the quantitative results. As noted, there was a tendency for junior and more experienced teachers to give qualitatively different responses. Veteran teachers commented more on systemic factors and more advanced aspects of medical education. In contrast, younger teachers expressed more basic needs, such as guidance in clerkship requirements, lesson planning and teaching methods. These results highlight young teachers’ struggles with even the most basic topics related to teaching, and stress the need for training before beginning their role as clinical teachers. This also underscores the changing needs of clinical teachers throughout their careers, and the need to adapt training to differing needs over the course of physicians’ careers.

The methodology of this survey subjects it to potential sources of bias. No mechanism was implemented to secure equal representation of the participating medical schools. The were significant differences between the Technion and the other 3 universities in rates, length and content of pedagogic training. In the Technion many physicians with no academic appointment received short pedagogic training. These caveats aside, the data provide a basis for understanding the needs of clinical teachers in Israel. In light of reports of the inadequacy of training clinical teachers from other regions [6, 11–13], the findings of our study can be extended universally. In the context of deprived resources plaguing medical systems, teaching training can empower physicians and improve their motivation and wellbeing [6]. We showed previously that allocating work time for physician empowerment can improve their performance and wellbeing [7]. Yet, the validity of our findings should be interpreted in relation to the absolute number of physicians that answered the questionnaire, and the proportion of replies that we received of the total number of invitations. The number of responses we received seems appropriate to the size of the Israeli medical system. The number of responses from the Technion Medical School was higher than the responses from other faculties, and this may represent a weakness in representation in our survey. The reply rate of 58% is similar to the rate of 59% reported by Cardemil et al. [46] and high, compared to the rate of 32% in a survey by Poncette et al. [47]. While internal medicine is the major and the lengthiest theme taught in all medical schools, the presentation of teachers from internal medicine was relatively low in the survey.

The findings of our survey should serve for planning the future of medical education in Israel. The major issues that should be addressed are inadequate training and the content of training, and the recognition and rewards of teaching medical students in academic promotion and in material benefits. These issues were also raised by the international committee which performed an external review of the medical schools in Israel in 2014 [14, 15] (for full report see appendix). Although some progress can be seen for example by the recent initiative of the Israel Medical Association with courses for physicians to be trained as medical educators and the medical educator track for promotion in some of the medical schools, a major change is needed as echoed in our survey. This change will come about only if all involved parties recognize its need, starting with the Council of Higher Education (MALAG), the universities, Israel Medical Association and finally the faculty members of the faculties of medicine. Resources should be allocated for pedagogic training including clinical (beside) teaching of physicians and the recognition of the importance of clinical teaching by all faculty members should be acknowledged and rewarded. In view of the expectations of young physicians as expressed in our survey, it is our concern that without a major change, poorly motivated teachers and low quality teaching will result in poorly educated young physicians and irreversible damage to the Israeli medical system.

## Conclusions

Several issues arising from the results of this survey require attention.

The first concerns training doctors for the teaching role, as most physicians involved in clinical teaching in Israel do not receive any training in this domain. There were significant changes between the Technion and other faculties of medicine in some findings but a common theme was that the training seems inadequately tailored to the needs of those who do receive it. Together with the problem of lack of specialized and adequate training for clinician-educators, medical teaching is perceived as inferior to medical research. To maintain motivation in teaching, the importance of teaching and of its status as an essential academic activity must be recognized. Further, appropriate rewarding for quality teaching must be addressed. In order to improve medical teaching and medicine itself for the next generation this study should prompt change in the way physicians are trained to teach, and are valued and rewarded for teaching.

## Supplementary Information

**Additional file 1: Table S1**. Self-Confidence and Improvement in Self-Confidence. Scale: 1 – not at all, 5 – to a very great extent. **Table S2**. Motivation of physicians to be involved in education. Scale: 1 – not at all, 5 – to a very great extent. **Table S3**. Important attributes of a clinical teacher. “To what extent are the following attributes important in a clinical teacher who is a role model?”. Scale: 1 – not at all, 5 – to a very great extent. **Table S4**. Appreciation and reward for medical education. Scale: 1 – not at all, 5 – to a very great extent. **Table S5**. Sub-analysis of responses to the question “I believe that good teaching is appropriately rewarded and appreciated”, according to the experience of the respondents and the timing of the teaching. Scale: 1 – not at all, 5 – to a very great extent. **Table S6**. Sub-analysis of attitudes to the statement “In my opinion, improving appreciation and/or compensation for medical education would result in improved teaching for medical students”, according to the experience of the respondents and the timing of the teaching. Scale: 1 – not at all, 5 – to a very great degree. **Table S7**. Sub-analysis of responses to the question “I feel that, relative to involvement in research, involvement in medical education is better (scale: 5) / similarly (scale: 3) / more poorly (scale: 1) rewarded and appreciated” by variables. **Table S8**. Representative examples of responses to the open question “What are the aspects of greatest importance in your opinion regarding the training of doctors for teaching in the clinical environment?” broadly categorized by theme. *n* = 126.

**Additional file 2.**

## Data Availability

Data are available upon request.
