# Supplementary material for: Survey of faculty development in four Israeli medical schools: clinical faculty development is inadequate and clinical teaching is undervalued in Israeli faculties of medicine
Source: Isr J Health Policy Res. 2021 Feb 8;10:10. doi: 10.1186/s13584-021-00438-0 (PMC7871531; doi:10.1186/s13584-021-00438-0)
Supplement: Supplementary file 1 — Additional file 1: Table S1. Self-Confidence and Improvement in Self-Confidence. Scale: 1 – not at all, 5 – to a very great extent. Table S2. Motivation of physicians to be involved in education. Scale: 1 – not at all, 5 – to a very great extent. Table S3. Important attributes of a clinical teacher. “To what extent are the following attributes important in a clinical teacher who is a role model?”. Scale: 1 – not at all, 5 – to a very great extent. Table S4. Appreciation and reward for medical education. Scale: 1 – not at all, 5 – to a very great extent. Table S5. Sub-analysis of responses to the question “I believe that good teaching is appropriately rewarded and appreciated”, according to the experience of the respondents and the timing of the teaching. Scale: 1 – not at all, 5 – to a very great extent. Table S6. Sub-analysis of attitudes to the statement “In my opinion, improving appreciation and/or compensation for medical education would result in improved teaching for medical students”, according to the experience of the respondents and the timing of the teaching. Scale: 1 – not at all, 5 – to a very great degree. Table S7. Sub-analysis of responses to the question “I feel that, relative to involvement in research, involvement in medical education is better (scale: 5) / similarly (scale: 3) / more poorly (scale: 1) rewarded and appreciated” by variables. Table S8. Representative examples of responses to the open question “What are the aspects of greatest importance in your opinion regarding the training of doctors for teaching in the clinical environment?” broadly categorized by theme. n = 126. [file 13584_2021_438_MOESM1_ESM.docx]

Supplementary Table 1 – Self-Confidence and Improvement in Self-Confidence.
Scale: 1 – not at all, 5 – to a very great extent.

| Topic | Mark the most appropriate option regarding the extent of your self-confidence in the topic: (n=245) | | Mark the most appropriate option regarding the increase in your self-confidence following pedagogic training: (n=86) | |
| --- | --- | --- | --- | --- |
|  | Mean | SD | Mean | SD |
| Giving feedback | 3.89 | 0.76 | 3.39 | 1.27 |
| Assessing learning | 3.75 | 0.8 | 2.99 | 1.23 |
| Using varied teaching methods | 3.71 | 0.81 | 2.95 | 1.28 |
| Lesson planning | 3.88 | 0.82 | 2.54 | 1.26 |
| Pedagogic theory | 2.97 | 1.10 | 2.22 | 1.14 |
| Guidance regarding course contents | 3.21 | 1.05 | 2.25 | 1.31 |
| Guidance regarding the required level of knowledge in the course | 3.78 | 0.87 | 2.45 | 1.26 |
| Guidance on setting examinations | 4.24 | 0.69 | 2.93 | 1.32 |
| Guidance on preparing educational materials | 4.19 | 0.74 | 3.01 | 1.28 |
| Principles of bedside teaching | 4.15 | 0.7 | 2.95 | 1.19 |
| Small group teaching | 3.84 | 0.9 | 2.99 | 1.26 |
| Discussing clinical challenges | 3.51 | 1.02 | 2.56 | 1.2 |
| Communication skills | 3.9 | 0.82 | 2.7 | 1.15 |
| Discussing ethical challenges | 3.89 | 0.76 | 3.39 | 1.27 |
| Role modelling | 3.75 | 0.8 | 2.99 | 1.23 |
| The training improved my educational capabilities (n=86) |  |  | 3.14 | 1.0 |

Supplementary Table 2 – Motivation of physicians to be involved in education.
Scale: 1 – not at all, 5 – to a very great extent.

|  | Mean | SD |
| --- | --- | --- |
| I feel motivated to be involved in teaching students (n=245) | 4.41 | 0.75 |
| My motivation to teaching stems from: (n=245) |  |  |
| A desire to contribute to the next generation of doctors | 4.50 | 0.64 |
| A desire to “pay back” my former teachers | 2.97 | 1.22 |
| The understanding that teaching is an integral and important part of being a doctor | 4.47 | 0.68 |
| The understanding that teaching improves my learning | 4.52 | 0.72 |
| The sensation that teaching gives me energy | 4.02 | 1.07 |
| The desire to please my superiors | 2.38 | 1.15 |
| Requirements for an academic appointment | 2.84 | 1.30 |
| Financial reward / prizes | 1.70 | 0.95 |
| The training that I received regarding medical education increased my motivation to be involved in teaching (n=86) | 2.93 | 1.18 |

Supplementary Table 3 – Important attributes of a clinical teacher. “To what extent are the following attributes important in a clinical teacher who is a role model?”
Scale: 1 – not at all, 5 – to a very great extent.

| Attribute | Mean | SD |
| --- | --- | --- |
| Deep knowledge in the relevant medical field | 4.61 | 0.54 |
| Superior clinical thinking | 4.72 | 0.49 |
| The ability to communicate clearly | 4.71 | 0.51 |
| High motivation to learn | 4.67 | 0.53 |
| Empathic | 4.58 | 0.63 |
| Inspirational | 4.66 | 0.56 |
| Love of his/her work | 4.71 | 0.54 |
| Respectable appearance | 3.81 | 0.91 |
| Superior interpersonal communication | 4.65 | 0.53 |

Supplementary Table 4 – Appreciation and reward for medical education.
Scale: 1 – not at all, 5 – to a very great extent.

|  | Mean | SD |
| --- | --- | --- |
| I believe that good teaching is appropriately rewarded and appreciated (n=245) | 2.81 | 1.17 |
| In my opinion, improving recognition and compensation for medical education would result in improved teaching for medical students (n=245) | 4.11 | 0.84 |
| I feel that, relative to involvement in research, the compensation and appreciation of involvement in medical education is better (scale: 5) / similarly (scale: 3) / more poorly (scale: 1) (n=245) | 1.33 | 0.91 |

Supplementary Table 5 – Sub-analysis of responses to the question “I believe that good teaching is appropriately rewarded and appreciated”, according to the experience of the respondents and the timing of the teaching.
Scale: 1 – not at all, 5 – to a very great extent.

| **Variable** | **Mean** | **Significance** |
| --- | --- | --- |
| **Medical Specialty** | | |
| Internal Medicine (n=123) | 2.80 | NS |
| Obstetrics & Gynaecology (n=45) | 2.67 |  |
| Paediatrics (n=77) | 2.91 |  |
| **Holds an Official Academic Appointment** | | |
| Yes (n=76) | 2.71 | NS |
| No (n=169) | 2.85 |  |
| **Received Training for Clinical Teaching** | | |
| Yes (n=86) | 2.98 | NS |
| No (n=159) | 2.72 |  |
| **Medical School** | | |
| Ben Gurion University of the Negev (n=28) | 3.04 | P=0.000 |
| Hebrew University of Jerusalem (n=29) | 1.9 |  |
| Technion Institute of Technology, Haifa (n=104) | 2.72 |  |
| Tel Aviv University (n=84) | 3.15 |  |
| **Years of Educational Experience** | | |
| 0-2 years | 2.98 | NS |
| 3-5 years | 2.66 |  |
| 6-10 years | 2.85 |  |
| Over 10 years | 2.65 |  |
| **Stage of Professional Development when Completing the Survey** | | |
| Junior Resident (prior to Part 1 exam) | 2.93 | NS |
| Senior Resident (following Part 1 exam) | 2.95 |  |
| Junior Specialist (less than 5 years’ experience as a specialist) | 2.72 |  |
| Senior Specialist (more than 5 years’ experience as a specialist) | 2.70 |  |

Supplementary Table 6 - Sub-analysis of attitudes to the statement “In my opinion, improving appreciation and/or compensation for medical education would result in improved teaching for medical students”, according to the experience of the respondents and the timing of the teaching.
Scale: 1 – not at all, 5 – to a very great degree.

| **Variable** | **Mean** | **Significance** |
| --- | --- | --- |
| **Medical Specialty** | | |
| Internal Medicine (n=123) | 4.12 | NS |
| Obstetrics & Gynaecology (n=45) | 4.07 |  |
| Paediatrics (n=77) | 4.10 |  |
| **Holds of an Official Academic Appointment** | | |
| Yes (n=76) | 4.14 | NS |
| No (n=169) | 4.09 |  |
| **Received Training for Clinical Teaching** | | |
| Yes (n=86) | 4.15 | NS |
| No (n=159) | 4.08 |  |
| **Medical School** | | |
| Ben Gurion University of the Negev (n=28) | 4.21 | NS |
| Hebrew University of Jerusalem (n=29) | 4.24 |  |
| Technion Institute of Technology, Haifa (n=104) | 4.13 |  |
| Tel Aviv University (n=84) | 3.99 |  |
| **Years of Educational Experience** | | |
| 0-2 years | 4.23 | NS |
| 3-5 years | 4.11 |  |
| 6-10 years | 4.13 |  |
| Over 10 years | 4.00 |  |
| **Stage of Professional Development when Completing the Survey** | | |
| Junior Resident (prior to Part 1 exam) | 4.15 | NS |
| Senior Resident (following Part 1 exam) | 4.03 |  |
| Junior Specialist (less than 5 years’ experience as a specialist) | 4.21 |  |
| Senior Specialist (more than 5 years’ experience as a specialist) | 4.04 |  |

NS: nonsignificant

Supplementary Table 7 - Sub-analysis of responses to the question “I feel that, relative to involvement in research, involvement in medical education is better (scale: 5) / similarly (scale: 3) / more poorly (scale: 1) rewarded and appreciated” by variables.

| **Variable** | **Mean** | **Significance** |
| --- | --- | --- |
| **Medical Specialty** | | |
| Internal Medicine (n=123) | 1.31 | NS |
| Obstetrics & Gynaecology (n=45) | 1.27 |  |
| Paediatrics (n=77) | 1.42 |  |
| **Holds an Official Academic Appointment** | | |
| Yes (n=76) | 1.29 | NS |
| No (n=169) | 1.36 |  |
| **Received Training for Clinical Teaching** | | |
| Yes (n=86) | 1.47 | NS |
| No (n=159) | 1.26 |  |
| **Medical School** | | |
| Ben Gurion University of the Negev (n=28) | 1.36 | NS |
| Hebrew University of Jerusalem (n=29) | 1.21 |  |
| Technion Institute of Technology, Haifa (n=104) | 1.33 |  |
| Tel Aviv University (n=84) | 1.38 |  |
| **Years of Educational Experience** | | |
| 0-2 years | 1.47 | NS |
| 3-5 years | 1.36 |  |
| 6-10 years | 1.34 |  |
| Over 10 years | 1.23 |  |
| **Stage of Professional Development when Completing the Survey** | | |
| Junior Resident (prior to Part 1 exam) | 1.49 | NS |
| Senior Resident (following Part 1 exam) | 1.24 |  |
| Junior Specialist (less than 5 years’ experience as a specialist) | 1.31 |  |
| Senior Specialist (more than 5 years’ experience as a specialist) | 1.17 |  |

NS: not significant

Supplementary Table 8 – Representative examples of responses to the open question “What are the aspects of greatest importance in your opinion regarding the training of doctors for teaching in the clinical environment?” broadly categorized by theme. n=126.

| **Systemic issues that influence clinical teaching** | |
| --- | --- |
| Time must be allocated for teaching within the health care system. In Israel, there is inadequate recognition of the needs of teaching. | Department Chair, 10+ years teaching |
| The first thing to emphasize is teaching residents. If there is no teaching in the department, there will also be no proper student instruction. If the manpower is inadequate for routine [clinical] roles, there will be no teaching in the department. One can’t expect a team to both perform routine daily tasks and to succeed in teaching effectively. | Junior Specialist, 6-10 years teaching |
| Dedicated time should be included in doctors’ schedules specifically for this purpose | Junior Specialist, 6-10 years teaching |
| There is no organizational memory except within specific departments for clinical rotations, with each department reinventing the wheel. And, of course, there is no standardization between departments… There should be a position that deals with standardization and organizational memory – in the Faculty of Medicine, the Office of the Deputy Dean in the hospital… but in practice it does not happen. | Senior Resident, 10+ years teaching |
| Creating an organizational culture of teaching as an inseparable part of a doctor's job, regardless of his or her area of specialization. | Senior Specialist, 6-10 years teaching |
| The performance of training [for teaching] and a system that supports teaching within the framework of the hospital and the university for all departments of the hospital / doctors involved (budgeting, defining this as the role of a hospital deputy director, etc.) | Junior Specialist, 3-5 years teaching |
| Today, teaching is an integral part of the work day. When there are not enough residents, there is no one to teach the students in an orderly manner. There is not enough planning on the subject. Dedicating teaching time, and increasing clinical staffing in wards, in the understanding that clinical instruction is critical, may improve the situation. | Senior Resident, 3-5 years teaching |
| Employing additional doctors will enable some to dedicate themselves to education and teaching students during their stay in the department. Appropriate training of doctors involved in teaching students. | Senior Specialist, 10+ years teaching |
| **Remuneration and recognition of clinical teaching** | |
| Much more significant weight must be given to teaching within the criteria for academic promotion | Department Chair, 10+ years teaching |
| Recognition by the academic system of the importance of teaching by academic and financial reward (conferences, appointments, etc.), at least to the same extent as research. | Junior Specialist, 3-5 years teaching |
| **Institutionalization of clinical educator training and the establishment of goals and standards in clinical clerkships** | |
| Investing resources and finding ways to increase motivation for teaching from the early student stage, and involving students and residents in teaching.  Establishment of a (compulsory) course on pedagogy, either in medical school or as part of specialty training. | Department Chair, 10+ years teaching |
| Defining the goals of clinical teaching - that is, the doctors involved in teaching teach "as they see fit". Perhaps more uniformity is required in this regard, in terms of the material the faculty wants to be passed on to the students.  Not every department is "teaching-friendly" and sometimes students suffer as a result. Before sending students to a particular department, it is best to clarify with the department head what the requirements are from the tutor - both in terms of the material being taught, and in terms of the amount of time that needs to be made available for teaching purposes. | Senior Specialist, 2-3 years teaching |
| If teaching is considered an integral part of the doctor's role, such classes should be taught in medical faculties – no differently than anatomy.  Only by investing in the younger generation will we be able to improve our future. | Junior Specialist, 6-10 years teaching |
| Providing information about the students’ level and setting clear goals regarding the purpose of teaching (what students are expected to know, etc.) | Senior Specialist, 3-5 years teaching |
| [Clearly outlining] the requirements of the course, and coordination of the needs and expectations of the university and the students | Senior Specialist, 0-2 years teaching |
| **Desirable content in the training of teacher physicians** | |
| Instruction regarding preparing study materials, preparing lesson plans and tips on training at the bedside -- because we did not receive enough training before we started our teaching careers. | Senior Resident, 0-2 years teaching |
| Tutor-student relations and how to deal with various types of barriers to learning | Junior Resident, 0-2 years teaching |
| Teaching methods both frontal and bedside – emphases and differences between the two methods. Lesson planning, etc.  Writing lesson plans, preparing presentations | Senior Resident, 0-2 years teaching |
| Lesson structure and conveying messages  Interpersonal communication | Senior Resident, 3-5 years teaching |
| How to teach in different ways - small groups/one-on-one, exams, lectures, etc. | Junior Resident, 0-2 years teaching |
| Alertness to the various types of students and how to bring them closer to the bedside in fields that are not of interest to them.  How to turn clinical rounds into something interesting and interactive, and remove the feeling of it being an examination but rather inspiring learning. | Junior Specialist, 3-5 years teaching |
| How to be a mentor / role model | Senior Specialist, 10+ years teaching |
| Interpersonal communication, including providing feedback, increasing motivation to learn  Diversity of teaching methods | Senior Resident, 0-2 years teaching |
| Learning how to make clinical thinking and processing accessible to students  Learn how to give feedback and motivation to independent student learning | Junior Resident, 0-2 years teaching |
| Understanding the different natures of students, and adapting, accordingly, methods that may facilitate active learning | Senior Specialist, 10+ years teaching |
| Clinical lesson demonstration / guided ward rounds by experts. | Junior Specialist, 0-2 years teaching |
| **Personal traits of a clinical teacher / qualities to be passed on to students** | |
| In my opinion, it is very important to convey humility, modesty and interpersonal communication, and to demonstrate empathy and understanding of the patient. | Junior Specialist, 6-10 years teaching |
| The key to good teaching lies, in my view, in a genuine desire to be a teacher. Beyond that, there are "additional requirements," including: having the knowledge to be taught, being competent as a teacher, and a willingness to invest [in teaching] regardless of remuneration | Senior Resident, 3-5 years teaching |
| A desire to teach and empower the future generation  Love of the profession | Junior Specialist, 3-5 years teaching |
| Ability to discuss clinical cases effectively and to build a clear clinical approach.  Motivation to teach, to be attentive to students and to identify flaws in their thinking | Senior Resident, 0-2 years teaching |
