# Supplementary material for: Survey of faculty development in four Israeli medical schools: clinical faculty development is inadequate and clinical teaching is undervalued in Israeli faculties of medicine
Source: Isr J Health Policy Res. 2021 Feb 8;10:10. doi: 10.1186/s13584-021-00438-0 (PMC7871531; doi:10.1186/s13584-021-00438-0)
Supplement: Supplementary file 2 — Additional file 2. [file 13584_2021_438_MOESM2_ESM.pdf]

Committee for the Evaluation of Medical Study Programs

General Report

August 19, 2014

Contents

Chapter 1: Background……………………………………………………………..……..3

Chapter 2: Committee Procedures………...…………………….………………..……..5

Chapter 3: General Report…………………………………………………………........…6

Chapter 4: Summary of Recommendations…………………………………......44

Appendices: Appendix 1 – Letter of Appointment……………………………...51

Appendix 2 – Dates of the visits……….……………………………....52

Appendix 3 – Council for Higher Education Standards for Evaluation of Medical Schools and Medical Education…………………………………..53

Chapter 1- Background

The Council for Higher Education (CHE) decided to evaluate the study programs in the field of Medicine during the academic year of 2014.

Following the decision of the CHE, the Minister of Education, who serves ex officio as Chairperson of the CHE, appointed a Committee consisting of:

- Prof. Stephen Schoenbaum – The Josiah Macy Jr. Foundation, New York, USA: Committee Chair[1]
- Prof. Raymond H Curry – Northwestern University Feinberg School of Medicine, Illinois, USA
- Prof. Shimon Glick - Professor emeritus in medicine, Faculty of Health Sciences, Ben Gurion University of the Negev, Israel [2]
- Prof. Peter Crome - Department of Primary Care and Population Health, University College London, United Kingdom
- Prof. Elliot Gershon, Department of Psychiatry and Behavioral Neuroscience, University of Chicago, Illinois, USA
- Prof. David Katz – Professor Emeritus of Immunopathology, Faculty of Medical Sciences, University College of London, United Kingdom
- Prof. Ora Paltiel, Attending Physician, Department of Hematology- Hebrew University Hadassah Medical School, Hebrew University; Professor of Epidemiology, Braun School of Public Health[3]
- Prof. Jo Shapiro – Harvard Medical School, Harvard University, Massachusetts, USA

Ms. Daniella Sandler- Coordinator of the Committee on behalf of the CHE.

Within the framework of its activity, the Committee was requested to:[4]

1. Examine the self-evaluation reports, submitted by the institutions that provide study programs in Medicine, and to conduct on-site visits at those institutions.
2. Submit to the CHE an individual report on each of the evaluated academic units and study programs, including the Committee's findings and recommendations.

---

1 Prof. Schoenbaum's concomitant position as chair of the external International Advisory Review Committee (IARC or Goldman Committee) to Ben-Gurion's University's (BGU's) Faculty of Health Sciences was reviewed by the CHE prior to his appointment, and deemed not to be in conflict with his role as chair of the current committee.

2 In accordance with the CHE's policy, Prof. Shimon Glick did not participate in the evaluation of BGU to prevent the appearance of a conflict of interests.

3 In accordance with the CHE's policy, Prof. Paltiel-Clarfield did not participate in the evaluation of BGU or in the evaluation of Hebrew University-Hadassah Faculty of Medicine (HUJI) to prevent the appearance of a conflict of interests.

4 The Committee's letter of appointment is attached as **Appendix 1**.

3. Submit to the CHE a general report regarding the examined field of study within the Israeli system of higher education including recommendations for standards in the evaluated field of study.

The entire process was conducted in accordance with the CHE's Guidelines for Self-Evaluation (of October 2012).

Chapter 2 - Committee Procedures

The Committee held its first meetings on 23 February, 2014, during which it discussed fundamental issues concerning higher education in Israel, the quality assessment activity, as well as medical study programs in Israel.

During February and March 2014, the committee conducted full-day visits at Ben-Gurion University of the Negev (BGU) and the Technion. In May, 2014, committee members visited Hebrew University-Hadassah (HUJI) and Tel Aviv University (TAU). Then, in early June, 2014 four members of the Committee visited the Bar Ilan University medical school campus in Tzfat.[5]

During the visits, the Committee met with various stakeholders at the institutions, including management, faculty, staff, and students.

What follows is the general report that the Committee was also requested to prepare in its initial charge.

The dates of the visits to the various schools are listed in **Appendix 2**.

[5] The four members visiting BIU were Profs. Crome, Glick, Katz, and Schoenbaum. The Committee was cordially invited by BIU for this visit, but it was not part of the initial formal charge of the Committee. The four visitors did prepare an informal report for the School's rector and dean.

Chapter 3: General Report

Introduction:
The Committee's visits to the medical schools gave it an opportunity to appreciate many fine features of the universities in Israel and their faculties of medicine/health sciences. These include:

- The applicants to medical schools are academically at the top of all Israeli students and that almost all the students have a *sechem* score that is in the top two percent of the country. It is quite impressive that the best students are choosing to pursue careers in medicine.
- At each institution, the students generally are very happy with the education they are receiving and with entering the medical profession.
- At each school, the faculty members that we met are seriously committed to their educational roles.
- There is an impressive level of research in all of the institutions.
- These very positive features exist in spite of many budgetary and organizational problems within the schools that could potentially affect both faculty and students.

Thus, it is within this context that the Committee hopes its comments, even its critical comments, will be understood to be entirely constructive in their intent.

The Committee wants to express its appreciation for the extensive self-evaluation information we had received for each school and for the hospitality extended to us everywhere we visited.

Organization of the report:
The Committee has organized this report in parallel with its reports on each of the schools it was initially charged to visit. What follows are sections related to the following:

1. Mission and Goals
2. Organizational Structure
3. Study Programs
4. Human Resources / Faculty
5. Students
6. Teaching and Learning Outcomes
7. Research
8. Infrastructure
9. Evaluation Process (national and local)
10. Additional Important Policy Considerations

The Committee makes recommendations at the end of each section and aggregates them in Chapter 4. In contrast to our practice in each of the individual reports, we do not specify a timeframe for each of the recommendations. We know that some

recommendations can lead to short term processes and that others are likely to extend into a longer period; but, we think that all are of high priority and should be initiated within the year of receipt of this report and completed as soon as possible. Each recommendation should lead to a project plan that sets out the elements of a suitable process to address the particular issue and creates a schedule for the individual elements and the overall process.

Also, in contrast to our reports for the individual schools, and despite the length of this report, we have not developed an executive summary. Instead we list the major issues raised in each section in the following table:

Section 1, Mission:

- Stating clearly the primary obligation of each school to develop physicians as competent clinicians, and stating other elements of each school's mission
- Consistently reflecting the School's mission in the study programs of the school and in the execution of study programs
- Integrating professionalism into the study program of each medical school and reflecting this commitment in the school's mission.

Section 2, Organizational structure:

- Having elected deans
- Meeting the Council for Higher Education's Standards for Evaluation of Medical Schools and Medical Education.
- Issues related to having a Faculty of Medicine that encompasses schools in multiple health professions

Section 3, Study programs:

- Resource competition by English language programs designed for non-Israelis with the Hebrew language programs for a growing number of Israeli students
- The length of the overall trajectory for educating a physician (initial schooling, the stage, residency). Whether it is sufficiently effective and efficient
- Collaboration between medical education and health care providers
- A systems approach to the preclinical years

Section 4, Human Resources/Faculty:

- Having as prerequisites for faculty promotion demonstrating proficiency in teaching proficiency and demonstrating professionalism
- Revision of the promotion process to value excellent educators and clinical innovators
- Faculty development and remediation

Section 5, Students:

- Efficient use of faculty in the admissions process

- Potential conflict between the need for students to earn money while in medical school and their deriving maximal benefit from their education and clinical exposures.

Section 6, Teaching and Learning Outcomes:

- Articulating appropriate overall learning outcomes (competencies) and then objectives for each course or clerkship in the curriculum
- Aligning methods of teaching and assessment with learning objectives (increasing active learning, increasing assessment of competence vs. just knowledge)
- Developing ambulatory medical education
- Increasing clinical exposure and responsibilities, starting early in the educational process
- Integrating the preclinical and clinical curricula
- Supporting faculty development with strong centers for medical education

Section 7, Research:

- Collaboration and research
- Having a broad approach that includes educational research and social science
- Issues associated with the MD-PhD programs
- Postdoctoral training
- The MD thesis

Section 8, Infrastructure:

- Study centers vs. traditional libraries
- Central/national resources

Section 9, Evaluation process:

- Strategic planning
- Changing world standards related to accreditation of medical schools

Section 10, Additional Important Policy Issues:

- Understanding national health care needs and using them as a basis for workforce planning
- Aligning the financing of medical education with developing the workforce the nation needs
- Achieving collaboration between the educational system and the health care delivery system
- Renewing and enhancing the primary care workforce

Many of the issues are interrelated, and some are mentioned in multiple sections. We hope that readers will be able to navigate the text that follows.

1. Mission and Goals:

Most medical schools around the world have a tripartite mission involving excellence in teaching, clinical care, and research. This mission reflects the understanding that there are many different career pathways within the medical profession, and medical schools must be capable of producing physicians who have the required competencies for entering one or more of these careers. Most physicians will engage in clinical practice during some portion of their careers. In addition, some physicians will become researchers in either the basic or clinical sciences; some will enter public health. Yet despite these varied career pathways, most schools acknowledge that their primary obligation is to produce physicians who have acquired the competencies essential for careers in clinical medicine, which includes many different possible choices of specialty and subspecialty.

Indeed, the above is also reflected in the Israeli Council for Higher Education's Standards for Evaluation of Medical Schools and Medical Education which states at the outset, "The main objective of a program of medical education leading to the M.D. degree in Israel is to prepare its graduates to enter and complete graduate medical education, to qualify for licensure, to provide competent medical care, and to have the educational background necessary for continued learning. A medical school may establish additional objectives for its educational program, consistent with its program resources. A medical school must define its objectives and make them known to faculty and students." It is both desirable and understandable that individual schools may have different overall goals, orientations and emphases. These should find expression both in their missions and be reflected consistently in their programs.

The Committee formally reviewed four medical schools. There are differences among them in terms of tradition and location that no doubt impact on how they define their mission. The Committee felt that at the Technion, there was insufficient clarity of mission. This appeared to contribute to insufficient consistency between some of the statements of the mission and the content of the study program. Specifically, the Committee felt that the Technion, while a laudable institution and highly respected institution for technology and science education, had not satisfactorily addressed the part of its mission relating to the development of future clinicians. The Committee also felt that at Hebrew University, although the mission was clear, it had not been sufficiently communicated to guide a study program based on developing competencies. Indeed, in all four of the schools, even when desired competencies have been stated clearly (e.g., Tel Aviv University), the curricula and learning methods employed are not specifically competence-based (see below, Teaching and Learning Outcomes).

In addition, because the Committee believes it is incumbent upon medical schools to foster professionalism in the broadest sense for all of its graduates, we recommend that professionalism be woven into the study program for each medical school and

that this commitment be reflected in each school's mission. Professionalism is based on knowing how to build and support trustworthy relationships. As such it is not a separate topic or limited teaching project; nor can its acquisition be blindly assumed.

The Committee observed that within each medical school's preclinical curricula professionalism was introduced though some teaching in the humanities - often in association with an effort to introduce students to what it is like to be a practicing physician. These learning opportunities appear to be valued by both students and faculty. For the most part, however, they are confined to the preclinical years despite intentions by some schools to extend them into the clinical curriculum. Furthermore, teaching in the humanities does not necessarily satisfy the need for discussion of issues related to professionalism as part of the curriculum. In some schools, the Committee learned that students feel that there is no time to discuss professionalism issues when they arise during the clinical years.

The Committee believes that development of physicians into highly competent professionals needs to be explicitly taught, modeled and assessed throughout the entire educational experience. Because many professionalism dilemmas are contextual, the teaching faculty should provide opportunities to discuss and learn from real life tensions in the practice of clinical medicine and research. While each institution can decide the specific content and delivery of the professionalism framework, it should include the following components, each of which should be integrated into all years of medical education:

- Honesty and integrity
- Respect for patients and families
- Interpersonal communication skills including respectful communication with all healthcare team members
- Altruism and social justice
- Self-reflection and commitment to lifelong learning

The Committee has noted in the individual school reports its specific concerns about the mission statements or about the way in which the stated mission is aligned with other elements of the school, particularly the study program.

Recommendations:

- Consistently reflect each school's overall goals in both the school's mission statement and within the content of its study program.

- A commitment to fostering professionalism among its administration, faculty and students should be stated in the mission of each medical school and reflected throughout the School's activities. (also see Section 4, Faculty)

2. Organizational Structure

Each Faculty of Medicine or Faculty of Health Sciences that the Committee visited has an elected dean with the exception of the new Bar-Ilan University (BIU) Faculty of Medicine in the Galilee (Tzfat) where the founding dean has been appointed for a five year term. Subsequent deans at BIU will be elected for 4-year, non-renewable terms. At Ben-Gurion University of the Negev (BGU), the dean is elected for a three year term and eligible for re-election once; but as events since the Committee's visit in February 2014 have demonstrated, this eligibility for re-election at BGU requires the consent of the rector. The dean at Hebrew University-Hadassah (HUJI) is elected for a four year non-renewable term, and there is a rotation of the deanship between a preclinical faculty member and a clinical faculty member. At the Technion, the dean is elected for a two-year term but is eligible for re-election twice. Generally, however, deans do not serve more than four consecutive years. The dean at Tel Aviv University (TAU) is elected for an initial five- year term and may then be reelected for one additional three-year term.

The Committee's visit to TAU occurred at almost the end of Dean Mekori's eight years in the position. We noted the great progress that has been made there since the report of the CHE committee in 2007, shortly after he took office, and attributed that to a combination of his personal qualities and his continuity in office.

Overall, however, the Committee has several concerns about elected deanships involving medical study programs.

- Election to deanship must be from the internal faculty. Thus, there is limited opportunity for an elected dean to bring the potential benefits of significant outside experience to her/his role.
- The elected deans must be full professors; and although we shall discuss appointments and promotions in Section 4, Human Resources/Faculty, it is important to note here that scientific publications are the most important determinant of promotions in each of the institutions the Committee visited. That will result in deans who have significant experience and knowledge of scientific research but may not lead to the availability of candidates who have significant experience and knowledge of health care delivery, medical education, the other health professions, or education in other health professions.
- Election is by definition a political process. The electorate is unlikely to vote for a person who intends to create radical change even if the institution needs such change. To put it another way, a candidate often is led to make promises that are not ideal for the institution in order to line up the votes needed to get elected.

- Governance issues that require a central university committee or officer's approval, e.g., appointments, promotions, and in some instances curriculum changes, can thwart a dean whose term is short from achieving needed changes even when his/her faculty members favor them. This is particularly a problem at the Technion.
- At HUJI, TAU, and the Technion, there is a rotation of the deanship between persons representing clinical and basic sciences. This can be another limitation to picking the best candidate for the times and situation, which should be a top priority. The main mission of a medical school is to train physicians, and there are undoubted advantages of a physician serving as a role model for medical students. This should be one of the factors that contributes to the decision making process about medical school leadership. Notably, physicians are the deans in the overwhelming majority of medical schools in the Western world.

Given all the above, the Committee believes that none of the medical schools can guarantee consistently meeting the requirements in section 3, Administration, of the CHE's Standards for Evaluation of Medical Schools and Medical Education (see Appendix 3) as they relate to the dean.

At three of the four schools the Committee visited, i.e., all except the Technion, the Faculty of Medicine or Faculty of Health Sciences, encompasses more than medical study programs. For example, at Ben-Gurion University of the Negev (BGU), in addition to two medical "schools", the Joyce and Irving Goldman Medical School and the Medical School for International Health, and other medical study programs leading to masters and doctoral degrees in the medical sciences, the Faculty of Health Sciences encompasses the School of Pharmacy, the School of Medical Laboratory Sciences, and the Recanati School for Community Health Professions. The last has three component programs: nursing, physical therapy, and emergency medicine. The corresponding Faculties at Hebrew University (HUJI) and Tel Aviv University (TAU) include a somewhat different array of components.

Although the charge to the Committee was to review medical study programs in Israel rather than all of the activities of the Faculty of Medicine or Faculty of Health Sciences of a university, the organizational structure of these Faculties raises considerations that should be important to CHE and the administration of each institution offering MD degrees.

To the best of the Committee's knowledge, the elected dean of the Faculty in each institution has always been an MD or PhD in the medical sciences, not a nurse, pharmacist, etc. The Committee wonders if the interests of those not in medicine or the medical sciences are appropriately represented and attended to during the term of the dean?

Having multiple health professions schools aggregated in one faculty may have some advantages. For instance, it should be easier to develop and implement interprofessional education (IPE) programs. Several countries believe that fostering better teamwork among health professionals is essential for meeting the health care needs of the population more effectively and efficiently; and developing better teamwork can be facilitated by IPE. The World Health Organization (WHO) definition of IPE, is the following: "Interprofessional education occurs when students from two or more professions learn about, from and with each other to enable effective collaboration and improve health outcomes."[6] Although in the Faculties that encompass multiple schools it is common for faculty members to teach students in more than one school and sometimes the learners from different schools are together in the same classroom, that situation does not meet the WHO definition. Only one of the schools the Committee visited (BGU) has made a formal effort to do interprofessional education (IPE).

Another potential advantage is facilitating development of resources that can support educational efforts in more than one school and resources that can be shared across schools. Sharing of lecture rooms and laboratory facilities does occur. Sharing simulation facilities is another possibility. Indeed, not only can simulated settings be used effectively for education of several different types of health professionals but simulation has also been a rich environment for IPE.

Recommendations:

- CHE should review each of the Committee's individual reports in relation to section 3 of its own Standards for Evaluation of Medical Schools and Medical Education.

- CHE needs to review and define clearly the roles of the Deans of Medical Schools, taking into account not only their responsibilities for research and education tasks, a role common to that of deans of other Faculties, but also their professional training roles in medicine and other professions if the Faculty encompasses more than one profession. Given the uncertainty about the capability of an electoral process and short timescale to deliver suitable candidates, it may be that an appointment process or an extended term of elected office may serve better than the present system to ensure that all medical schools have appropriate chief academic officers.

- Require each university that has a Faculty of Medicine or Health Sciences encompassing students in different health professions to submit a report on how its governance effectively represents the needs of each and all of its

[6] See: WHO Framework for Action on Interprofessional Education & Collaborative Practice; White Paper 2010; http://whqlibdoc.who.int/hq/2010/WHO_HRH_HPN_10.3_eng.pdf

types of students, how it is or is planning to develop interprofessional education (IPE), and how it already has, or plans to have, resources that can be used by multiple schools to enhance the effectiveness and efficiency of their educational programs.

3. Study Programs

All of the institutions the Committee reviewed have multiple study programs leading to an MD degree. All but the new BIU school have a 6-year Hebrew language study program consisting of three preclinical and three clinical years. BIU has a 3-year program for persons who have already had the equivalent of the three preclinical years at another school usually in another country. Both TAU and BIU have a 4-year Hebrew language program for Israelis who have already received a B.Sc. degree. HUJI has a second 6-year Hebrew language program (Tzameret) to develop physicians for the military. BGU, TAU, and the Technion all have 4-year English language programs for foreign students. BGU's 4-year English language program, the Medical School for International Health, emphasizes global medicine and accepts students from multiple countries. TAU and the Technion's 4-year English language programs are strictly for North Americans.

As noted in the individual school reports for BGU, TAU, and the Technion, our Committee received much less information about the 4-year English language programs than about the 6-year Hebrew language programs. There are several important issues associated with these medical study programs that merit consideration:

Standardized comparative evaluations of the students enrolled in, and the educational effectiveness of, the English-language and Hebrew-language programs have not been done. Such evaluations would assure the quality and comparability of the two types of program.

The English-language programs are in resource competition with the programs for Israeli students in a several ways.

First, there is one pool of faculty members in each institution to draw from. Over the first decade of the new millennium, there has been a diminution in the size of the preclinical faculties owing to decreased funding to the universities. Since the Israeli and non-Israeli students cannot be combined in a classroom due to different language skills, there is now more competition for faculty than at the beginning of the 2000s.

The number of Israeli students has been increasing in each 6-year program in accordance with the government's perceived needs for more Israeli-trained physicians. The increase in numbers of students has not been

accompanied by a corresponding increase in staffing. We discuss later the desirability of decreasing current dependency on frontal lectures and increasing the use of interactive teaching methods. Should this occur, there will be an even greater demand for a limited number of faculty members.

Another source of resource competition is the limited availability of hospital teaching beds. Three types of medical students need to be accommodated: the Israeli students in 6-year or 4-year programs; the non-Israeli students in the Israel-based English language programs in Israeli institutions, and a cohort of students from schools in other countries that are being offered clinical clerkships in Israel. Everywhere, the Committee heard that hospital resources have become increasingly stretched and that priorities and limits need to be set.

Increasing ambulatory teaching has a high value on its own and potentially offers an alternative to a significant amount of hospital-based teaching. To date, there has not been sufficient development of the ambulatory sector for teaching. So, this too is a limited teaching resource. The CHE committee that evaluated medical schools in 2007 said, "We conclude that the CHE, or another national agency, will have to play a major role in designing and implementing the collaboration between medical education and the health-care providers needed for optimal ambulatory health-care teaching."[7] The current Committee notes it did visit two community teaching clinics that are joint efforts of BGU and Clalit. Since we were not offered similar experiences in other medical schools, we believe the recommendations of the CHE committee in 2007 have yet to be implemented

The existing English-language programs provide money that is separate from the funding the institutions receive from VATAT. The Committee did not receive evidence, but it was implied that this money subsidizes other programs. Hence, if the English-language programs are discontinued, an alternate source of funding would be necessary.

In light of all these issues associated with the existence of multiple study programs within medical schools, in particular the English-language programs for non-Israelis in addition to the 6-year and 4-year Hebrew language programs for Israelis, the Committee feels it is important that there be a national assessment of the plusses and minuses of authorizing Israeli institutions to have/continue the programs.

The charge to our Committee did not include review of the entire educational trajectory of an Israeli physician, e.g, a 6-year program, or a 4-year post-baccalaureate program, followed by a seventh year of rotating internship before the MD degree is conferred (the stage), followed by a 4-5 year post-graduate residency

---

[7] Committee for the Evaluation of Medical Schools in Israel, General Report. October 2007. Chapter 4.2.4., p.8. Available at: http://che.org.il/wp-content/uploads/2012/04/The-General-Report-2.pdf

program – a total of 11 to 14 years. It is important not only to consider the effectiveness of the full trajectory but also its efficiency.[8] In the U.K., the core undergraduate program is six years, but most medical schools exempt one year resulting in a five year course of study.[9] Preclinical education is usually systems-based and there is significant clinical exposure from early in the program. This is followed by a two year "foundation programme" and then 4-7 years of "specialist training", with general practice regarded as a distinct specialty. In the U.S., the usual course of study includes four years of college leading to a baccalaureate degree, four years of medical school leading to the M.D. degree, and then 3-5 years of residency. Preclinical education in medical school is systems-based, and clinical education, which starts early, is highly experiential.

Thus, in both the U.S. and U.K. the educational trajectory is no longer, and in some instances may be shorter, than in Israel. The U.S. and U.K. medical schools have re-evaluated their programs in recent years, introducing desirable features that are not present in all the schools in Israel, and are regarded as desirable for 21st century physicians. These include systems-based preclinical education, significant clinical exposure from an early stage in the program, and considerable supervised and observed clinical experience. In addition, in the U.S., particularly owing to the cost of medical education and the degree to which graduates are in debt, there is now discussion about whether the trajectory can be shortened, and there are a few 3-year undergraduate medical education programs.

In contrast to the U.S. and U.K. study programs, the Committee felt that in Israel there was excessive teaching of basic science information that was not relevant to students' future careers and that in the clinical years there was too little direct clinical exposure and responsibility. These are discussed further in Section 6, Teaching and Learning Outcomes.

Recommendations:

- CHE should charge a work group with formally assessing the degree to which the English-language medical school study programs compete for scarce resources that are needed for meeting national needs. The scope of the work group should also include assessment of the revenue vs. costs of the English-language programs and the degree to which funding to the universities and their medical schools would need to increase should these programs no longer exist.

[8] The Committee addresses this issue more fully in Section 10, Additional important policy issues.
[9] In England and Wales it is only Oxford, Cambridge, University College London, and Imperial College London that have a six year course. These study programs also incorporate a BSc or BA degree.

- Review/assess the educational effectiveness of the English language programs as compared with the Hebrew programs, particularly for clinical education where language barriers could be significant.[10]

- There should be a formal assessment of the seventh year (stage) as part of the undergraduate education of a physician.

- To enhance the effectiveness and efficiency of medical education there should be a work group that formally assesses the overall trajectory of educating a physician in Israel and analyzes alternatives to the current one. This is likely to require examination of alternative teaching methods and teaching sites (e.g., ambulatory sites). It should be accompanied by assessments of both educational outcomes and costs.

- A systems approach to the early years of medical education, the "preclinical years", should be encouraged in all medical schools as should collaboration in that teaching by basic science and clinical faculty members.

- The 2007 recommendation that "CHE or another national agency play a major role in designing and implementing the collaboration between medical education and health-care providers" should be broadened so that it is not just about ambulatory health-care teaching but about all the resources needed for effective medical and other health professions education. The current Committee is not aware that the 2007 recommendation was implemented across all schools and urges that it be implemented now.

4. Human Resources / Faculty

Each of the institutions we visited has a relatively small group of basic scientists whose promotions are largely dependent upon research productivity as measured primarily by the number and impact of their publications. These faculty members were reported to be responsible for most of the teaching in the first three years of the 6-year curricula as well as having some teaching responsibilities in various other professional schools that are part of some of the Faculties of Medicine or

[10] The Committee heard varying accounts about the adequacy of Hebrew language abilities for the students in the English-language programs. It felt their language instruction might not be enough for sophisticated interactions with patients and staff and particularly for reading any handwritten records. It wondered if their somewhat limited language skills might affect their learning in clinical settings and their subsequent performance in clinical settings when they return to programs in English-speaking countries. Perhaps this is one factor that accounts for the fact that students in Israeli medical schools who take the USMLE (U.S. Medical Licensing Examination), Steps 1 and 2, do not score as well as U.S. medical graduates. Another possible factor is that the vast majority of students from the U.S. who do undergraduate medical education in Israel were not accepted in U.S. medical schools and tend to have lower MCAT (Medical College Admission Test) scores.

Health Sciences, e.g., nursing, pharmacy, etc. There were some significant issues associated with these faculty members:

- With the small number of available basic science faculty and the large and increasing number of students in medical programs plus other programs, almost all the teaching has been done by frontal lectures and almost all the evaluation has been multiple-choice tests. These issues will be discussed in Section 6, Teaching and Learning Outcomes.
- The limited number also means that basic scientists who may be excellent researchers but not excellent teachers are nonetheless involved in teaching. The teaching load also can decrease available time for doing research. Since the existing promotions systems favor published research and not teaching excellence, basic science faculty members do not have a direct incentive to improve their abilities to teach.
- Difficulty in finding classically-trained PhD pharmacologists, anatomists, and experts in some other fields that are covered in traditional basic science programs has led to younger PhDs being assigned to teach in fields in which they are not expert.
- Difficulty providing teaching in laboratory medicine disciplines linked to the clinic, e.g., histopathology, microbiology, poses a problem: the academic posts for clinically qualified staff are few, and these posts are regarded as preclinical, but delivery in any format other than frontal lectures is dependent heavily upon the goodwill of hospital based practitioners who do not have academic status.
- Although it varies, in some schools there is little teaching by clinical faculty in the first three years of medical school. Clinical faculty could augment the available teaching staff for basic sciences - especially the clinically-relevant basic sciences - and also enable teaching the preclinical subjects in a systems approach.

Each of the universities/medical schools has worked out arrangements with hospitals and to a variable extent, some free-standing ambulatory care facilities, and is dependent upon a large number of professionals located in those hospitals and facilities to conduct clinical teaching. Of the clinical teachers, only a minority has faculty appointments. Those appointments are also primarily related to research and publications rather than to teaching abilities. This Committee feels that there would be benefits to the universities, the health care delivery system, and the country to develop a more inclusive process for clinical teachers both in clinical and non-clinical settings. As an example of the latter, surgeons in various disciplines and radiologists can participate in teaching anatomy, and a variety of clinicians in fields such as cardiology, infectious diseases, nephrology, and gastroenterology among other, can participate in teaching disease-related pharmacology. Encouraging interaction of basic scientists and clinicians can also facilitate the development of systems learning.

In the U.S. the rule, rather than the exception, is that physicians associated with major teaching facilities have faculty appointments at the affiliated university even though the role of that physician may be primarily in delivering clinical care. This ensures that the medical staff of that teaching facility to whom medical students and physicians who are still in training (internship and residency) meet a certain standard of quality. The appointments are generally associated with an expectancy that the physician will engage in a certain amount of teaching and various academic committee obligations. There are multiple tracks for promotions depending upon the area of academic concentration of the faculty member. For example, promotions at Harvard Medical School are designed for faculty whose academic concentration is teaching and education, clinical expertise and innovation, or investigation. All promotions at Harvard involve an expectation of excellence, scholarship, and a "link between the rank of the appointment and the breadth of the candidate's reputation." Scholarship at Harvard is no longer defined just as peer-reviewed publications but may include "educational materials, policy statements, assessment tools, and guidelines for patient care."[11] Although these U.S. comparisons are linked to a 4-year medical education program that may not be considered directly comparable to the Israeli system with its 6-year program, the U.K. experience of "medical academic" appointments, where a similar combination of teaching/education, research, and service, is recognized in joint medical school and healthcare provider employment contracts, does provide the necessary educators for such 6-year programs.

Faculty development is an essential function in educational institutions and certainly in medical schools. In general, the Committee feels that there are too few staff to assist faculty in improving their teaching and adopting newer methods of teaching and broader methods of assessment (subjects covered in more detail in Section 6, Teaching and Learning Outcomes). The resources and their activities vary from school to school and even department to department. For example, at TAU there is a simulation-based faculty development program for tutors involved in teaching the Ob/GYN clerkships. In most instances, the limited staff qualified to help faculty improve their teaching skills runs programs that are voluntary for faculty, even those who receive poor teaching ratings from students, and are focused on frontal lectures and multiple-choice tests. In a couple of instances, highly qualified staff had left the institution and had not been replaced or the positions were still vacant at the time of our visit.

Recommendations:

- Every school should have a policy that each physician who is hired to teach medical students and residents should have already demonstrated excellent professional values, understand that part of the job is to infuse professional

[11] As examples see the criteria for promotion at Harvard Medical School and the University of Chicago School of Medicine: Harvard - http://facultypromotions.hms.harvard.edu/promotions.pdf ; Chicago - https://webshare.uchicago.edu/users/vvv1/Public/Pathways_pdf.pdf

values in all contacts with learners, and be aware that demonstrated professionalism is a requirement for eligibility for promotion.

- CHE should encourage each medical school to have a promotions process that explicitly considers the quality of teaching performance for any faculty member who is expected to teach. Implementing this recommendation will require each school to develop suitable, auditable assessments of teaching and professional performance. Furthermore, even those faculty members whose promotion is under consideration primarily because of research excellence should have demonstrable skills in teaching that are satisfactory or better.

- CHE should encourage each university to develop a promotions process for its Faculty of Medicine or Health Sciences that values the multiple types of academic excellence and scholarship necessary for effective medical and health professions education. In addition to having a track for promotion that values traditional forms of investigation, there should be tracks that value teaching and educational scholarship in the preclinical and clinical areas, and scholarship related to clinical or programmatic innovation.

- CHE should encourage each Faculty of Medicine or Health Sciences to create a portfolio of faculty development and remediation activities and demonstrate that faculty performance is improving in all domains of medical education. Faculty development should also be facilitated by the new national organization of medical educators and having it open to all interested in medical education practices and research. There will need to be sustainable support for such an organization. (see Section 6, Teaching and Learning Outcomes)

5. Students

For its 6-year programs, each of the medical schools now has a two component admissions process. The first component addresses scholastic achievement through a combination of the matriculation and psychometric test results. The second component is related to personality and judgment, the objective being to find students who not only are scholastically excellent but also have good humanistic qualities.

For the second component, BGU has two faculty members interview the students using a process it developed at its inception in 1974. For many years it appeared that BGU's graduates were academically equivalent to the graduates of other Israeli medical schools but were more humanistic. Now, all of the medical schools except BGU use a standardized multi-station/interview process developed in Israel by the National Center for Medical Simulation (MSR) and based on the multiple mini-

interview (MMI) process created several years ago at McMaster University in Canada. The MMI and the process at MSR use trained persons, but not faculty members, to do the standardized interviews.

Not all the schools in Israel use MSR. At HUJI, faculty members have been trained to do the interviews; and the Committee was told that a study has demonstrated that the process at HUJI obtains similar results to those at MSR. HUJI feels that it is important to both faculty and potential students that they interact during the admissions process.

The Committee has some concerns about the current variation in admissions processes. First, it is not known if BGU achieves similar, better, or poorer results than MSR in assessing humanistic qualities of applicants. Second, since there is more than one process for implementing the multiple interviews, despite the reportedly comparable results to date, there is a potential for deviation from the standardized process over time. Most importantly, the Committee is concerned about the time required for faculty to engage in the processes at BGU and HUJI. Though it understands the desire to engage faculty with students, it also understands that there are many demands on faculty time. Lack of faculty availability is cited as a reason for not adopting more interactive teaching methods, performing assessments other than multiple-choice tests, and the like. That means there is an opportunity cost to not using MSR universally for this part of the admissions assessment. We have recommended both to BGU and HUJI that each evaluate the benefits and costs of its admissions processes and compare those to one using MSR.

The non-Israeli members of the Committee were surprised by the fact so many students have jobs while in medical school. Some of these jobs are potentially relevant to future careers in medicine, e.g., being a physician assistant or working in a laboratory; but students also reported choosing jobs that were not medically relevant because they pay more. The Committee is concerned that when a student has a job, s/he may not have sufficient time for class, clerkships, or studying. It does appear that the need to take jobs to earn money while in medical school curtails the exposure of students to clinical practice, both in a formal and informal way.

The Committee cannot state that students should not have jobs during medical school, especially if there is a financial need. If students are to be permitted to work, their jobs should be relevant to their future careers and pay sufficiently to meet financial necessities. Furthermore, it should be possible to structure physician assistant jobs for students, ensure that supervisors have formal teaching responsibility, and even develop a formal curriculum. This approach should help make these not just a job but an educational opportunity.

A very different issue involving students relates to how they best learn. The Committee was told in more than one school that Israeli students want to be "spoon-fed" and to learn by rote rather than engaging in active learning. The

students, however, noted that currently teaching is by lecture, and the bottom line is doing well on the multiple-choice exams. So, not surprisingly, they are focused squarely on the answers to those examination questions. Although not entirely without controversy, current educational theory favors active learning techniques and argues that retention of information is better. There are a couple of experiments in interactive preclinical courses at BGU and HUJI; and students uniformly reported a positive reaction to these.

Another opportunity to enhance the learning environment does not require interaction during class but involves frequent formative assessment, with prompt feedback of the test results and an opportunity for the student to learn from his/her failures and successes. Unfortunately, the Committee heard mostly about the lack of good feedback on tests at various medical schools.

Recommendations:

- CHE should have a work group evaluate the degree to which students in Israeli medical schools need to take jobs for financial reasons, and if there is significant financial burden consider policies that would alleviate that burden. The work group could also consider the ways in which jobs for medical students could best be structured to complement their medical education.

- Committee recommendations regarding teaching and assessment methods that enhance student learning can be found in Section 6, Teaching and Learning Outcomes.

6. Teaching and Learning Outcomes

The articulation of overall desired educational outcomes, or competencies, has become a fundamental expectation of accrediting bodies for medical education, including the World Federation on Medical Education (WFME).[12] Some, such as the Liaison Committee on Medical Education (LCME) in the U.S., further specify the need for linkages between these overall curricular objectives and specific learning objectives in each course or clerkship and for linkages to blueprints for course-level examinations. These linkages are essential to self-directed learning, the assurance that the knowledge and skills necessary for practice are being meaningfully

[12] The World Federation on Medical Education uses the terms "educational outcomes," "learning objectives" and "competencies" interchangeably. Others more often refer to competencies and educational outcomes as general, over-arching desired end points, and learning objectives when referring to the more detailed subordinate aspects of a particular learning experience.

assessed, and ultimately the assurance that medical graduates will be capable of providing high quality and safe patient care.

Only two of the four schools (TAU and HUJI) provide overall educational outcomes or competencies for their curriculum. In neither case are these general competencies explicitly mapped to course and clerkship objectives or to student assessment blueprints. At BGU and the Technion there is no global effort to coordinate and communicate learning objectives or student assessments in the manner described above. At the course/clerkship level, the Committee found isolated examples of very effectively constructed programs. The obstetrics and gynecology (OB/GYN) clerkship at the Technion, designed by clinical faculty along with consultation by an external expert in medical education, provides a good example. For the most part, however, learning objectives at the course/clerkship level at all four schools are superficially stated and do not serve as appropriately specific guides for meaningful student learning.

All the schools are cognizant of the need to move away from dependence on traditional frontal lecture-based curricula and provide a more active learning environment and opportunities for students to have a clinical context for learning early in medical school. There have been a few notable successes in the creation of learner-centered small group and discussion formats in preclinical curricula; but these are few and far between. Each of the Israeli programs is still very dependent on frontal lectures and offers much less opportunity for interactive and self-directed learning than medical school curricula in the U.S. In clinical education, the Committee also found the student role in the daily work of the department to be more passive and observationally oriented than is the norm in the U.K. and U.S. Clinical rounds and other activities for Israeli medical students are often separate from the activities of interns, residents, and faculty. While it is important to have some learning activities specifically directed to the student level, the students also need to learn active patient management principles by participating in the daily work of the clinical teams.

Another concern is the continuing paucity of opportunities for ambulatory and primary care education. This was highlighted by the 2007 CHE review committee but has not led to action in most schools. Each school was able to decide who the Committee should meet among the clinical faculty and what facilities to show the Committee. The Committee was shown primary care clinics only at BGU; and few faculty members based mainly in ambulatory settings were asked to meet with the Committee.

There are several reasons for emphasizing ambulatory medical education:

- The prevalence of chronic conditions is growing in the population, and most of the care of persons with one or more chronic conditions is in the ambulatory sector.

- It is now possible to provide much care in the ambulatory sector that used to require hospitalization. Health professionals need to learn how to function in teams both in ambulatory and hospital settings and to coordinate their roles efficiently within and between teams and settings.
- In acute care settings the objective usually is resolving the acute problem or in performing the indicated test or procedure. Outcomes in ambulatory care settings and particularly in primary care settings are often more complex and include helping the patient achieve better health status and functional status. Adherence to treatment recommendations is a critical factor in achieving successful outcomes and requires learning how to establish a trusting relationship with the patient.
- In addition, with a limited supply of hospital resources for teaching and the desire of the government that Israeli medical schools increase their student numbers, it makes sense for Israeli medical schools and other health professional schools to develop teaching in the ambulatory sector.

While all primary care is ambulatory-based, primary care and ambulatory care are not synonymous. There are additional reasons for emphasizing primary care/family medicine education:

- The scope of family medicine is the "whole person", indeed the whole family, and not just limited to part of the body. Future physicians, even if they ultimately will enter a hospital-based specialty, should have ample exposure to this approach.
- Primary care physicians and practices follow patients longitudinally over time. Rather than time always being of the essence as it is in acute, emergency and hospital-based care, time can be both a diagnostic and therapeutic variable. In a primary care practice, learners, irrespective of their future field, have an unparalleled opportunity to acquire the ability to cope with the uncertainty of diagnosis and the vagueness of symptoms, whether ultimately diagnosed or not.
- A 2012 report from the Organization for Economic Cooperation and Development, or OECD, praises the current strength of Israel's primary care system, but raises a concern about the stability of that system. It states, "While Israel has benefited from a substantial migration of doctors, this has created a major challenge for the future as the cadre of older doctors heads towards retirement in coming years. Ensuring that future doctors and nurses choose to work in primary care ought to be focus of policy, alongside continuing to expand the number of chronic diseases covered by performance data on health care clinics."[13]

Clinical simulation is a powerful and important tool for teaching and assessing fundamental skills, including basic technical skills, more advanced procedural skills, interpersonal communication, and teamwork. Simulation not only allows an

[13] OECD Reviews of Health Care Quality: Israel. Executive Summary, Assessment, and Recommendations. 14 October, 2012. Available at: http://www.oecd.org/els/health-systems/ReviewofHealthCareQualityISRAEL_ExecutiveSummary.pdf

opportunity to practice and perfect skills, but to do so without the possibility of inconveniencing patients or harming them. Indeed, simulation is extremely useful in improving the competence of health professional students before they have direct patient experiences; and a major rationale for developing simulation methods and simulation centers is to improve patient safety. Though simulation centers have become near universal in U.S. and U.K. academic medical centers, there is little utilization of clinical simulation in the teaching of medical students in Israel. There is some at TAU, where the affiliated Sheba Medical Center houses the Israel Center for Medical Simulation (MSR), one of the most renowned clinical simulation facilities in the world, and where the Simultech center at Meir Hospital is used for some medical student teaching and faculty development. The Committee feels that more could be developed at each medical school and noted that while BGU has plans to build a modern simulation center, the project was stalled at the time of the Committee's visit.

There is general recognition of the need for curricular "integration," a term sometimes used to refer to the multidisciplinary coordination of material being taught during a given portion of the curriculum ("horizontal integration") and at other times referring to attempts to bridge the traditional divide between "preclinical" and "clinical" education ("vertical integration"). At BGU, a "spiral" curriculum design is attempting to promote both vertical and horizontal integration. The spiral design has been implemented to some extent in the preclinical years, but neither BGU nor any of the other schools has yet achieved sufficient horizontal or vertical integration. Prevailing organizational structures tend to make integration very difficult since the faculty in basic science departments and those in clinical departments are often employed by different entities and have very different expectations for their involvement in the medical school. The requirement for a separate baccalaureate degree at the completion of the first three curricular years is another impediment to integration or even effective collaboration between preclinical and clinical curriculum components. One potentially useful approach, recently adopted at HUJI and present at BGU, is the establishment of a single curriculum committee responsible for oversight of both preclinical and clinical education.

Assessment of students' knowledge acquisition in a traditional lecture-based curriculum is usually accomplished through multiple-choice examinations with practical exercises sometimes providing a component of the grade when there is a laboratory component. Given the small proportion of the curriculum in the Israeli schools that is delivered by means other than frontal lecture, there is limited use of other assessment methods at present. Where problem-based learning, team-based learning or other interactive small group formats can be implemented, they should be accompanied by structured individual assessments by the faculty, student self-assessment, and other means of tracking the development of students' interpersonal skills, decision-making skills, and professional behaviors, as well as their acquisition of knowledge.

In the clinical learning environment, most schools and departments provide adequate personal supervision and observation of students' history and physical examination skills, with additional feedback through regular review of patient write-ups. In several instances, such as the obstetrics and gynecology clerkship at TAU and several at HUJI, a clerkship-long assignment to a faculty tutor allows ongoing formative assessment. For the most part summative assessment at the end of clerkships is predominantly through multiple-choice examinations. There is little use of performance-based assessment strategies involving clinical simulation at any of the four schools, e.g., OSCEs (Objective Structured Clinical Examinations).

Faculties of medicine are by their nature large, heterogeneous, and engaged in a wide variety of teaching activities some of which, e.g., supervision of students on the clinical floor or teaching in the operating suite, may not be undertaken elsewhere in the university. The complexity of a medical curriculum, with interrelated courses each of which involves a number of faculty members, requires considerable collaborative faculty efforts and infrastructure from the school. Most medical schools now house an administrative unit dedicated to the development and support of faculty as educators, fostering excellence in teaching and appropriate training and support in curriculum development and assessment strategies. Many also provide important advances in medical education research.

The four schools reviewed by the Committee each have such an administrative unit and/or rely in part on broader university resources. Only TAU, however, appears to provide a level of support sufficient to meet the basic needs for faculty development. The other three schools have at one time had an active and effective Center for Medical Education, but have suffered losses of faculty and staff in recent years (Technion and HUJI) or are attempting to rebuild a program that was dormant (BGU). Thus, at three of the four schools there is presently a major gap in this critical academic function.

The Committee developed a strong impression regarding the possibility for a national infrastructure to support medical education. The five medical schools in Israel are in close geographic proximity. There would seem to be a number of opportunities for collaborative faculty development, curriculum development (particularly given that these are the only schools in the world teaching medicine in Hebrew), and development of student assessment tools and programs. The Committee considered that a national organization to provide a forum for persons interested in medical education and to promote medical education as an academic discipline would be valuable. Indeed, following its visits, the Committee learned that such an organization is being established and planning its first meeting. It commends this, hopes that such an organization might not only facilitate the development of a national infrastructure related to medical education and even to some unique Israeli innovations.

<u>Recommendations:</u>

- All schools must articulate appropriate overall learning outcomes (competencies) for their curriculum, and should utilize them as the basis for individual course and clerkship objectives and for assessments, with all learning activities and expected outcomes explicitly linked to specific learning objectives. This process might be facilitated through collaboration at the national level.

- Reduce dependence on frontal lectures, providing more opportunities for interactive learner-centered small group and discussion formats (problem-based and team-based learning methods, for example) and for learning in clinical settings, even early in the curriculum.

- Provide sufficient and appropriate opportunities for clinical education in ambulatory settings, and especially in primary care.

- Provide students with opportunities for more active involvement in patient care activities, in both inpatient and ambulatory settings.

- Reduce dependence on multiple choice examination formats for student assessment. Expand the use of performance-based assessments, using faculty observation, patient instructors, or other clinical simulation methods as appropriate. Wherever individual students or small groups have contact with a faculty member over a period of a few weeks or more, the student should receive formal, written performance feedback.
    - National examinations in clinical fields already exist but have only a multiple choice format. Each national examination should utilize additional assessment methods, e.g., an OSCE, to determine that the desired threshold of clinical competence has been achieved or exceeded by students.
    - Consideration also should be given to developing ways of assessing that students in all study programs, including the English-language programs, have achieved or exceeded the desired thresholds of competence.

- Each of the schools is underutilizing clinical simulation methods for teaching as well as performance assessment. Further resource development, from small task-trainers to whole-body simulators, and including patient-instructor/standardized patient methods, is needed. This is another area in which a nationwide collaboration, utilizing resources such as are already available at the MSR clinical simulation center, may be helpful.

- Work toward meaningful integration of the curriculum, both horizontal and vertical. Ensure appropriate central oversight and authority for curricular change. Consider changes to the baccalaureate degree requirements so as to

allow the introduction of clinical perspectives and skills in the early curricular years.

- Rebuild and invest in medical education centers to provide needed expertise in faculty development, curricular and instructional design, and assessment.

- Encourage collaboration at the national level in carrying out several of these recommendations, particularly in the areas of curricular development and performance assessment.

- Nurture the new organization dedicated to scholarship in medical education, support and sustain it, so that it can help in developing a national infrastructure for medical education.

7. Research

Each of the schools we visited emphasized its excellence in medical research; and, as noted in the Introduction to Chapter 3, the Committee is impressed overall by the strength of medical research in Israel's medical schools. The researchers we met were well-trained and productive.

There are some efforts to foster collaborative research. HUJI has had a collaborative research program for many years; and BGU has one that began just a few years ago and provides internal funding to projects on a competitive basis. Although some research is almost purely clinical and other research is almost purely basic science without known clinical application, the Committee had some concern that in some schools there seemed to be independent, potentially redundant research programs in the medical schools and their affiliated hospitals, and that there also were possibly missed opportunities for collaboration.

Though some of the Faculties include Schools of Public Health, population health research was not emphasized in our Committee's visits. The Committee understands that there are some very prominent Israeli programs in this area and was told that most medical student theses are population-oriented. It feels it is important that population-oriented research be a visible component of medical schools.

Medical education research was also not emphasized. Indeed, the Committee was concerned to find that schools that espouse the need for medical students to learn to be investigators have not applied principles of investigation to the core tasks of educating and assessing those students or of developing faculty as teachers, coaches, and clinical tutors.

In each school, the Committee met students and faculty involved in the MD-PhD program. Although both TAU and the Technion have been able to attract more students to their programs, 10 each in the year before the Committee's visit, in general, the medical schools would like to have more students choose the MD-PhD track, especially HUJI where only two students entered the program in the year before the Committee's visit. The MD-PhD programs vary significantly across the different schools. For instance, at HUJI typical support is only 2 years for the PhD research project. The student is expected to complete the project while s/he re-enters the fourth year of the clinical curriculum. In contrast, at TAU, the PhD part of this program offers three years' support with encouragement to extend to four years as needed, thus not requiring that the student who is returning to clinical studies continue working on his or her PhD thesis at the same time.

The Committee has serious reservations about the two-year PhD programs for MD-PhD students, and about the widely-held expectation that the student will complete his or her PhD thesis while transitioning back to clinical education. It appears to compromise, in an unacceptable way, both the research training and the clinical training of the students. In the rest of the world, and in Israel for non-MD-PhD stu-ents, a PhD normally takes at least 4 years, and the experience of preparing the thesis is an important part of the entire educational experience. Any medical student transitioning to the clinical years has a great deal of reorientation and adaptation to go through, and this is even more difficult for students returning to a clinical track after several years away from medical education.

The Committee feels that in addition to making each program as attractive as possible for potential applicants by assuring excellent financial support, graduate courses in science aimed at the research field of the student, mentoring, and an adequate time to do a significant project, there are other steps that might be taken to enhance these programs:

> Perhaps through compiling information about current and former students across the country who have chosen the MD-PhD program, it might be possible in the admissions process to seek out individuals who have characteristics that might predict a strong interest in research activities.
>
> Another approach is through altering the preclinical curriculum to encourage student inquiry and general interest in research. Highly successful research-oriented medical schools in the U.S. have reduced lecture hours drastically and changed the nature of their basic science teaching. Their teaching is much more interactive and oriented towards problem-solving activities.
>
> Having a strong postdoctoral experience is important for the development of both for new MD- and non-MD-PhDs. For a PhD to obtain a faculty position in Israel, a postdoc abroad is a requirement. For an MD-PhD, especially if the time to complete a PhD thesis has been short, a postdoctoral experience

provides an opportunity for remediation as well as a potentially successful scientific experience.

Currently, going abroad does not guarantee a position back in Israel. Furthermore, although the Committee heard differing accounts, going abroad with a family appears to be more difficult for women than men and is a potential disincentive for women to choose an investigative path. This could be addressed by guaranteeing all whose performance as students meets a certain standard of excellence that there will be a position back in Israel when they have completed a postdoctoral program. Another possibility, though not guaranteeing a position to all who go abroad for a postdoctoral program, would be to guarantee a position to those who, in their postdoctoral programs, meet pre-established performance standards such as publications in high-quality journals, presentations at international conferences, prizes, etc.

All of the medical schools require a thesis for students in their 6-year programs. The thesis is generally valued by students. The majority are population studies rather than laboratory research. The Technion has an interesting process in which the student's proposed research topic and subsequently the thesis itself are reviewed by an external reviewer. In all schools many of the thesis products are published. The Committee did feel that in each school, various aspects of the thesis programs could be improved and has made specific comments and recommendations in the individual school reports.

Recommendations:

- MD-PhD programs should only be authorized if they provide the expectation that the time allotted and supported for the PhD is a minimum of three years.

- The Dean's Forum in which all five Faculty of Medicine/Health Sciences deans participate should compare the MD-PhD programs and consider best practices and possible ways to attract a substantial and stable group of students to those programs to the degree that they meet a national need for physician researchers.

- The Dean's Forum should consider developing a uniform policy that can be recommended for adoption to each parent institution that would encourage persons who obtain MD-PhDs or PhDs in medical sciences at that institution to obtain appropriate postdoctoral training and experience.

- The Dean's Forum should compare the undergraduate thesis programs across the schools and attempt to assess their educational value for all medical students and find and spread best practices.

8. Infrastructure

Infrastructure has strong interactions with the abilities to teach, learn, and do research. Infrastructure includes not only bricks, mortar, and equipment, but also the capability to support teaching, learning and research often through specialized services. Although the Committee heard about and was shown some of the infrastructure at each of the schools it visited, it was not possible in the time available to evaluate carefully the infrastructure at each school.

With the advent and near-universal availability of computers, access to the internet, and broad resources now available online, the ability of persons seeking to communicate with each other and the ability to access a huge library of information have grown enormously. Most students and faculty in Israeli medical schools are able to access their institution's online materials for courses, online journals, and to a lesser extent, textbooks from just about anywhere. Where there are exceptions or opportunities for improvement, they have been noted in the individual reports.

This ability to access information has changed the roles of libraries and librarians. The former are being transformed into study centers and the latter into facilitators and teachers for accessing needed information effectively and efficiently. HUJI and TAU have developed impressive study centers. The Technion's library has been under reconstruction and we were not able to visit it. The library at BGU which is on the Soroka Hospital campus appears to be inadequate both in space and hours of operation for at least the needs of the students in the clinical years. There are some spaces for study for preclinical students in the area of the medical school.

The HUJI study center has an array of areas in which students can work individually, in small groups, or even large groups. This should facilitate team-based learning even though that method is not yet widespread in the curriculum. The HUJI study center staff also described to the Committee a highly student-centered approach to facilitating learning.

Each of the schools has lecture halls for its students, some of which have been renovated recently; and with some exceptions, noted in the individual reports, these seemed adequate for traditional frontal lectures. To some extent, even large lecture halls are usable for "flipped classroom" teaching and learning. A first step, already available in the medical schools in Israel, is the use of clickers to collect and assess student responses. Modern teaching methods also employ break-out rooms in which the learning is directed by the students and the role of the staff is to function as facilitators of student learning not instructors. We were unable to evaluate the degree to which adequate facilities are available for this process.

The Committee has already mentioned both the scarcity of hospital resources and the independent need for ambulatory teaching not just the fact that it can substitute for hospital-based learning. Assessing the equitability of the distribution of available hospital resources and the degree to which competing demands could be reduced is important.[14] Even more important is determining the degree to which ambulatory facilities would need to be altered to become suitable sites for education of medical students. This issue was also raised by the CHE committee in 2007.[15] , We are unaware of any systematic action to address it.

It may make sense to provide some needed elements of infrastructure centrally or to augment local school efforts with a central resource. As has been mentioned in Section 6, Teaching and Learning Outcomes, MSR is an existing resource that could play a broader role in undergraduate medical education nationally. It could help each medical school expand its uses of simulation to augment aspects of clinical medical education, including skills training, communication, and teamwork. It could also help expand the assessment of medical student performance and the assessment and improvement of teacher performance. This could be particularly useful in expanding ambulatory teaching where there is less opportunity for peer group interactions than in hospital teaching settings.

There also may be local resources that could be supported centrally so that their scope of activities could expand to cover all of the medical schools and possibly other health professional schools. For instance, at HUJI there is a multi-media center led by an individual with enormous experience making videos for teaching purposes. He has created a few thousand videos over the years. The existing collection of videos might have broader uses, and potentially one could develop the center into a national resource for creating needed videos. For example, in Section 6, Teaching and Learning Outcomes, we mention the possibility of developing courses or components of courses that could be used in all the medical schools that have such a course. Lectures on subjects that are considered essential to be offered to medical students in more than one medical school could be prepared by the best teacher of that subject. This could be one way to address the fact that in some schools basic scientists are currently being required to teach courses/subjects they feel unprepared to teach. It also could reduce the need for someone to give the same live lectures to each group of medical students in a clerkship several times a year. If these lectures were prepared professionally, they could be made available

---

[14] See Section 3, Study Programs, about evaluating the degree to which the English-language programs at Israeli medical schools compete with national needs for educating Israeli students and also the use of Israeli hospitals for teaching by non-Israeli medical schools.

[15] Committee for the Evaluation of Medical Schools in Israel, General Report. October 2007. Chapter 4.2.4., p.8. Available at: http://che.org.il/wp-content/uploads/2012/04/The-General-Report-2.pdf "…the medical schools will not succeed in developing adequate ambulatory teaching by merely changing the curriculum; the relevant health-care providers will have to agree to costly changes in clinic design and personnel deployment."

on the web, accessible by students when they need them. This would also reduce the need for live front lecturing and permit more innovative live teaching sessions.

In Section 6, Teaching and Learning Outcomes (Recommendations) we also have mentioned the desirability of having a national organization or forum for persons with an interest in advancing medical education. Such an organization, since it is being formed, should be a shared national resource to facilitate exchange of best practices and group development of improved approaches to teaching and assessment.

Recommendations:

- CHE should foster consideration of how existing national resources such as MSR could assist medical schools throughout the country in expanding their use of simulation in medical education.

- CHE should foster consideration of how existing local resources such as the multi-media program at HUJI could be helpful in medical education programs elsewhere in the country and how best to transform a local resource into a national one.

9. Evaluation Process (National and Local)

This is the third comprehensive review of medical education in Israel since 2000, the second since the establishment of the Council for Higher Education's Quality Assurance Division in 2003, and the first to be done by committee composed primarily of non-Israelis. The Committee understands that the goal of CHE in having committees composed of non-Israelis perform its quality assurance reviews is to obtain as objective an evaluation of programs and schools as possible. We hope we have been able to serve that purpose for medical education in Israel.

The Committee also applauds the fact that the longstanding CHE practice has been to foster transparency of review findings with reports posted on publicly accessible web sites. This provides an aspect of accountability and openness that would be unusual in many other countries.

Periodic external reviews should not substitute for internal and ongoing review and improvement by each medical school. In its individual reports the Committee has recommended that schools have an internal strategic planning process in which there is an environmental scan, critical self-assessment, and setting of strategic goals and specific strategies and tactics to achieve the goals. Such a strategic planning process, which is common in businesses, is also applicable to educational

institutions.[16] It should lead to periodic internal review every 1-2 years with updating of plans for improvement. Such a process also makes it easier to prepare for periodic external reviews.

The Committee was impressed by aspects of such planning at TAU where initial preparation of self-evaluation report materials by the Faculty led to a "to-do" list and, in turn, to a documented set of actions some of the issues that were identified. We feel that this approach is consistent with the quality improvement intended by CHE and is a step towards an ongoing strategic planning, improvement, and review process.

As a result of having an international committee, it has been possible for the reviewers to compare current Israeli medical education and accreditation practices with those in the U.S. and U.K. This seems fortuitous especially since medical education practices are changing around the world and since the current review comes at a time when there also is a new international process for the recognition of national organizations that accredit medical schools: The World Federation for Medical Education (WFME) now has an explicit process that recognizes national accreditation organizations as meeting their criteria for appropriate oversight of medical education. Since the inauguration of this program in 2012 and as of this writing, only the Liaison Committee on Medical Education (LCME) in the US and two other accreditation authorities, in Turkey and in the Caribbean, have been recognized by WFME, but momentum in other countries is growing.[17]

It is in a spirit of being constructive that we offer the following comments about the implications of WFME recognition of national organizations that accredit medical schools and of the present CHE system for oversight of medical education in relation to the new WFME process. Indeed, we believe that Israel needs to react promptly to the WFME's new recognition process:

> Israeli medical education enjoys a high international stature and reputation that is dependent upon its comparability to best practices elsewhere in the world. In the future that will also require that its medical school accreditation processes are recognized by the WFME and considered to meet an international standard.
>
> A common, indeed nearly universal, practice for aspiring Israeli academic physicians has been to pursue advanced training in international settings. This has helped ensure comparability of Israeli medical education to that in other countries. It also has generated important collaborations for Israeli

---

[16] For an example, see the University of Toronto Faculty of Medicine's strategic plan: http://www.facmed.utoronto.ca/Assets/FacMed+Digital+Assets/Faculty+of+Medicine+1/FacMed+Digital+Assets/misc/strategicplan.pdf

[17] See http://www.wfme.org/accreditation/accrediting-accreditors/recognition-process for details on the WFME recognition process.

physicians. But in order to take the USMLE examinations that are a prerequisite for training in the U.S., medical graduates must first be credentialed/certified by the Educational Commission on Foreign Medical Graduates (ECFMG). This applies to Israeli citizens seeking advanced fellowship training and also to all students in the English-language programs of Israeli medical schools who intend to pursue residency training or practice at any point in the future in the United States. The ECFMG has declared that as of 2023 it will no longer certify applicants from countries that do not have a WFME-recognized accreditation process.[18]

If CHE fails to attain WFME recognition soon, there may be another implication for the American citizens studying in Israeli medical schools. Currently, CHE's quality assurance system meets eligibility requirements for federal educational loans from the U.S. Department of Education. It is possible, even likely, that these requirements will be made comparable to those of the ECFMG at some point in the future.

Since graduates of Israeli medical schools as of 2023 will have been admitted several years before then, the Committee feels it is important that Israel position itself now so that its medical school accreditation processes are recognized by the WFME.

The Committee sees several ways in which CHE processes differ from or fall short of best practices in other countries and/or the expectations of the WFME recognition process. Accordingly, the Committee is concerned that CHE will not achieve WFME recognition as an accrediting authority without changing some of its practices with respect to faculties of medicine.

The Committee was not charged with assessing the Israeli schools, individually or collectively, against specific WFME standards or those of other national accrediting bodies. Thus, this is not a comprehensive assessment since that would be outside both the scope of the Committee's assignment and the resources available to it. Nonetheless, we hope the following points provide convincing initial evidence of the need to proceed with systematic changes:

- The CHE Standards for Evaluation of Medical Schools and Medical Education are presented as general statements, rather than the explicit expectations found in comparable documents from the U.S. Liaison Committee on Medical Education (LCME) and the U.K.'s General Medical Council (GMC). As a result, the review committee does not have concrete standards for deciding whether or not a given school is in compliance or not in compliance with CHE expectations.

[18] See: http://www.ecfmg.org/about/initiatives-accreditation-requirement.html

- The CHE Guidelines for Self Evaluation are only a general outline for the content of self-review documents. Schools thus have considerable latitude in deciding what data are provided for review. Templates for standardized collection of data including student demographic and performance data, program evaluation data including student responses, and financial reports would lead to much more efficient and accurate review by the external review committees.

- There is no explicit CHE process for adverse accreditation actions and thus no true ongoing accreditation authority. Nor are there robust mechanisms to ensure that quality assurance issues noted by a review committee will be addressed. Indeed, we have noted in the individual school reports and in this general report the variable degree to which recommendations of the 2007 CHE committee for the evaluation of medical schools in Israel have been addressed.

- Once reports from the review committee are submitted, the CHE itself through its Sub-committee on Quality Assessment is responsible for the evaluation of the report, its adoption, and the determination of any subsequent follow up reporting requirements. In order to address the unique characteristics and demands of professional education in medicine, the approach in the U.S., and according to the best information available to Committee members, the expectation of the WFME, is to have the institutional accreditation process be led by peer-reviewed self-regulation from within the profession, under the overarching authority of governmental agencies. In the U.S., the LCME is sponsored by the Association of American Medical Colleges and the American Medical Association; and it is charged by the Department of Education with the authority to issue accreditation decisions. The Committee feels that CHE will need to construct and charge a professional body with the authority to accredit under ultimate auspices of the CHE.

<u>Recommendations:</u>

- The Committee strongly recommends that CHE adopt processes consistent with the WFME recognition guidelines for accreditation authorities, and do so in advance of the date (2023) when ECFMG will require such recognition. This will no doubt alter the expectations Israeli schools must meet for subsequent quality assurance review cycles. The definition of new accreditation standards and timetable for their implementation would follow once new overall processes are in place.

    In approaching this challenge, we suggest that CHE convene a committee representing appropriate stakeholders such as Israeli

medical professional organizations, medical education leaders, and governmental authorities, and also some international experts. The purposes would be to review current CHE policies and procedures in light of international expectations for accreditation of medical training and create a proposal to address these issues.

- WFME is including a parallel process related to postgraduate medical training and similar issues are applicable to other health professions overseen by the CHE. We recommend that CHE familiarize itself with these and address them as well.

10. Additional Important Policy Considerations

Medical education, in fact all health professions education, should be considered in the context of improving the health of the population. This includes improving the general health status of the population as well as helping each individual who is ill or has a chronic condition achieve the best possible outcome. Although other types of professional education, e.g., law or architecture, also have a societal context, medical education may be unique in having three strong components – professional practice, teaching, and research.

Unless a country has a central, ongoing planning and policy effort, it is unlikely that a balance will be struck between the health needs of the population and the attention and resources devoted to teaching, research, and patient care. Israel is no exception. The needs of the country should determine the nature of the overall health professional workforce, the funding to develop and sustain that workforce, and the contribution of each of several possible funding sources. The impression of the Committee is that there are mismatches in Israel between the present and projected future needs of the country and the emphasis placed by the medical schools on meeting those needs. The Committee understands the desire by the government for there to be more physicians educated in Israel, and that this has led to the development of the new Bar-Ilan medical school in the Galilee and increases in class size in the existing schools. As a result of the increases in medical school class size and the additional of a new school, each of the four older schools expressed concern to the Committee about the limited hospital resources available for teaching, an issue that has to be considered in future planning. Furthermore, Israel up to now has depended on having physician clinicians; whereas several other countries also have significant numbers of non-physician clinicians, e.g., nurse practitioners, and some have expanded the roles of other non-clinician health professionals such as pharmacists. Planning for the workforce needed to meet the health care needs of the country should consider all types of health professionals and not just physicians.

The Committee understands that in Israel the health workforce planning is under the aegis of the Ministry of Health (MOH), not the CHE, all stakeholders should be involved. With respect to the physician workforce, elements that should be considered include the

process of physician periodic reregistration, an evaluation of what is occurring in each specialty, the ages of the physicians and their likely duration of active practice, projected needs in each specialty and in each area of the country. CHE must have the data necessary for aligning the outputs of the educational institutions with the present and future societal needs for physicians and other health professionals.

In addition, Israel will not have an appropriate physician workforce or overall health professional workforce unless the funding of medical education and other health professions education supports its development. Funding of undergraduate medical education in Israel occurs through the general funding of the universities that have medical schools. The formula that VATAT has created for all forms of higher education has the following formula for its teaching component:

$$\sum_{i} (\boldsymbol{T}_{(i)} \times \boldsymbol{S}_{(i)} \times \boldsymbol{E}_{(i)}) \times \boldsymbol{F}$$

This teaching component is "a 'bottom-up' absolute model consisting of the following indicators:

- $S_{(i)}$ - Number of students by discipline and degree level (bachelors and masters degrees only).
- $T_{(i)}$ - Fixed tariff per discipline and degree level.
- $E_{(i)}$ - Graduation rate coefficient based on the number of graduates divided by the total number of students.
- F – Student to academic faculty ratio coefficient at the institute level – not per discipline or degree level (as of 2010/11).

The fixed tariff per discipline and degree level does provide a higher amount for medicine than the humanities, but it appears to be inadequate to meet the demands of a modern medical education. First, we describe some of the characteristics of a modern medical education and then we specifically examine elements of the teaching component of the VATAT funding formula.

After being static for a long time, medical education is now continuing to evolve. Over 100 years ago, a science-based model began to dominate medical schools in developed countries. The catalyst for this was the Flexner Report.[19] The roots of the science-based model came from the work of German medical scientists of the mid-nineteenth century, the standardized graduate-level medical courses in European universities, and then the medical education curriculum established by Johns Hopkins Medical School in the late nineteenth century. Though the evolution of medical education from a pure apprenticeship model to a curriculum that began with basic sciences and progressed to clinical experiences has led to important progress, in the past couple of decades there has

---

[19] Flexner A. "Medical Education in the United States and Canada: A Report to the Carnegie Foundation for the Advancement of Teaching." Published by the Carnegie Foundation for the Advancement of Teaching, Bulletin Number 4 (1910). Available at: http://www.carnegiefoundation.org/sites/default/files/elibrary/Carnegie_Flexner_Report.pdf

been increasing recognition that reforms in medical education are necessary to prepare physicians for meeting the needs of the public in the 21st century. In recognition of the 100th anniversary of the Flexner Report, the Carnegie Foundation for the Advancement of Teaching commissioned a new study leading to a 2010 report that issued a new call for reform of medical education.[20]

The authors of the 2010 report stated, “Fundamental change in medical education will require new curricula, new pedagogies and new forms of assessment.” Among the authors' recommendations are:

- To standardize learning outcomes and assess competencies over time. A focus on learning outcomes and milestones could end the time-based structure of medical school and residency.
- To strengthen connections between formal and experiential knowledge across the continuum of medical education, specifically by incorporating more clinical experiences earlier in medical school and providing more opportunities for knowledge-building later in medical school and throughout residency.
- To promote learners' ability to work collaboratively with other health professionals, such as medical assistants, nurses, pharmacists, physical therapists and social workers.
- To support learners' responsibility for quality of care, team performance and their own learning while providing skilled supervision.
- To make professional formation an explicit area of focus in medical education through strategies such as formal instruction in ethics and reflective practice, exploration of the role of the physician-citizen and establishment of more supportive learning environments.
- To cultivate a spirit of inquiry and improvement in learners and in health care teams; this spirit supports both innovations in daily practice that translate into better service to patients, system improvements and improved patient outcomes as well as the development of larger research agendas, new discoveries, and knowledge building.
- To be more intentional about our selection, development and support of teachers and medical educators.

Although not all medical schools in countries such as the U.S. and U.K. have yet designed or implemented all of the recommendations of the 2010 report, many were headed in that direction even before the report was released. A 2011 paper by Nara et al, based on visits to 35 medical schools in 12 developed countries around the world, reported that “most of the medical schools have introduced a tutorial system based on PBL or TBL and simulation-based learning.”[21] These methods help address issues such as cultivating a spirit of inquiry, learning to work effectively in teams, and acquiring and perfecting skill-based competencies.

---

[20] Cooke M, Irby DM, O’Brien BC. “Educating Physicians: A Call for Reform of Medical School and Residency.”   Jossey-Bass, Higher and Adult Education Series, 2010. Summary available at: http://www.carnegiefoundation.org/sites/default/files/elibrary/Educating_Physicians_Summary.pdf
[21] Nara N, Suzuki T, Tohda S. “The Current Medical Education System in the World.” J Med Dent Sci 2011;58:79-83. Available at: http://lib.tmd.ac.jp/jmd/5802/07_Nara.pdf

The Committee's overall impression is that undergraduate medical education in Israel retains an emphasis on factual knowledge acquisition and retention. Yet there is a broader range of competencies that complement knowledge and that a physician should, acquire in the course of her/his education.[22] It is possible that these competencies may vary somewhat from country to country, but the point is that knowledge is just one area of competence. The needs of the Israeli population from their physicians need to be assessed to determine the sills that should be acquired and mastered at the undergraduate level. The curricula of Israeli medical schools should be oriented to ensuring that graduates acquire the breadth of competencies needed by Israel from its physicians and do so efficiently.

Comparing the current situation in Israel's medical schools with the recommendations of the 2010 Carnegie Foundation Report and trends across the world, there are important problems that need to be addressed:

- The educational format in Israel, particularly the emphasis on frontal lectures and multiple choice examinations, does not promote inquiry or skill acquisition. We were told more than once by faculty members that Israeli students want to be spoon-fed information. Even if that were the preference, it should not be encouraged.
- In the absence of a significant amount of team-based learning and interprofessional education, there is little to prepare graduates for working in the future in teams.
- The emphasis on hospital-based clinical experiences for medical students does not align with the increasing prevalence of chronic conditions in the population. Most chronic conditions can and should be managed in ambulatory care and primary care settings.

The current funding formula used by VATAT relating to medical education, even assuming that the universities distributed it to discipline exactly in proportion to the formula, includes the element "F" = "Student to academic faculty ratio coefficient at the institute level – not per discipline or degree level (as of 2010/11)". We do not know those coefficients for each institution we visited, but it is likely that they reflect a large amount of frontal lecturing to sizeable groups of students and do not account for the needs for faculty for the small group formats that have become increasingly prevalent in modern medical education. In addition, since much of the clinical teaching of medical students in Israel is done by capable physicians and others who are not considered faculty of the educational institution, the VATAT formula appears not to reflect the overall cost of educating a physician in Israel today. We know that there have been significant issues in the funding of clinical medical education between hospitals and universities[23], greater

---

[22] Englander, R, Cameron T, Ballard AJ, Dodge J, Bull J, Aschenbrener CA. "Toward a common taxonomy of competency domains for the health professions and competencies for physicians." Academic Medicine. 2013;88(8):1088-1094. doi: 10.1097/ACM.0b013e31829a3b2b

[23] Note: The Committee is aware that Profs. Glazer, Israeli, and Katz prepared a report entitled "Financial Arrangements between Universities and Hospitals, 2011" that was published in February, 2012, by the National Institute for Health Policy Research. We were told that it is providing some guidance to

in some places than others but seemingly present to some degree everywhere; and we anticipate that similar issues, perhaps even greater ones will arise if and when Israel decides that more ambulatory medical education must occur to meet the needs of the population.

We have noted in section 4, Human Resources/Faculty, that there should be reconsideration of appointments and promotions for clinical teachers. Since in Israel a university appointment carries certain benefits such as funds for meetings and sabbaticals, reconsideration of this area will require reconsideration of funding faculty involved in medical education. The current funding model and the associated appointment and promotion policies for clinical faculty leads to a disincentive for collaboration let alone integration of basic scientists and clinical faculty and for addressing the chronic complaint raised by students and the 2007 CHE medical education review committee that much of preclinical education is not relevant to the medical students' future careers. Ultimately, what may seem to be a parsimonious model for funding higher education may lead to less effective and less efficient production of the physicians the country needs. Thus, determination of a funding model or formula for medical education should be based not solely on determining the costs but on assessing the benefits in relation to the costs.

Much medical research is richer when informed by, if not directed by, persons who understand clinical needs. Current policies throughout Israeli medical schools do not provide dedicated salary support to clinicians for time they spend doing research. Thus, the time spent by clinicians to do research, and publication of research is currently the main way a clinician can be promoted, is considered by many a "labor of love". The desire to have an academic title probably provides some incentive for some physicians to do research. Nonetheless, providing dedicated research time for physicians, which is equivalent to ensuring that physicians are compensated for time they spend on a research project, should be a better way to increase the amount of clinically relevant research and would treat physician researchers comparably to non-physician researchers.

Having raised these issues, the Committee recognizes that several government ministries to varying degrees are involved in the support of teaching, and/or research, and/or patient care. Ministries involved in supporting these activities interact with several important stakeholders such as the universities that have medical schools and other health professions schools; the hospitals, some of which the government owns; and the sick funds. Planning the health care workforce for the rest of the 21st century and appropriately funding its development and maintenance are activities that are likely to involve all of these entities.

A 2012 report from the OECD (Organization for Economic Cooperation and Development) raises an additional, very specific point related to Israel's planning and

---

universities and hospitals in their mutual discussions; but we were unable to obtain a translation or summary in English. The report in Hebrew is available at: http://www.israelhpr.org.il/e/107/&SearchClean=1

educating its physician and health professional workforce.[24] First it praises teamwork within the existing primary care system in contrast to a concern it raises about coordination of hospital with ambulatory care. The report states,

> Israel provides a good example of how to undertake reforms to strengthen primary care. Over the past decade and a half, policy makers and health plans have sought to reorganise doctors working in the community into teams. This has provided them with a platform to do things that other OECD countries are struggling to do, like regular monitoring of a patient's health indicators, delivering follow-up support after a visit to the doctor, and tailoring preventative advice to the specific needs of communities. Israel's primary health care clinics are held accountable through extensive data collection on their activities.

It goes on to note,

> While Israel has benefited from a substantial migration of doctors, this has created a major challenge for the future as the cadre of older doctors heads towards retirement in coming years. Ensuring that future doctors and nurses choose to work in primary care ought to be focus of policy, alongside continuing to expand the number of chronic diseases covered by performance data on health care clinics.

Our Committee understands that since the migration of many who currently provide primary care involved persons who had already been through undergraduate medical education, Israel neither had to incur the cost of that undergraduate education nor because of the existence of this workforce up to now had to face squarely the issues of replacing those retiring or ensuring its educational system will lead to enough graduates who will meet the primary care needs of a growing population. Other policies may be relevant as well including the fact that persons who go into family medicine in the periphery are not eligible for the bonus that persons who go into specialties in the periphery can get.

One more important issue that has significant implications for educational policies and practices is the importance of examining the entire trajectory of educating a physician.[25] The charge to our committee was to evaluate the first 6 years of medical education. But, if one is trying to develop an effective and efficient educational trajectory for physicians, then there must be an assessment of that overall trajectory. We have noted that the combination of 6 years of medical school, the stage, and then a 4-5 year residency seems longer than it need be. It also should be noted that the recommendations of the 2010 Carnegie Foundation Report were meant to apply both to medical school and residency programs. The first recommendation cited above, "A focus on learning outcomes and milestones could end the time-based structure of medical school and residency," merits close consideration. Pilot programs have begun in the U.S. (pediatrics, starting in medical school) and Canada (orthopedics, starting in residency) that allow a learner to progress at a rate determined by his/her acquisition of competencies, a major objective of these programs is shortening the overall time it takes for learners to acquire necessary

---

[24] Kumar A, Colombo F, Abi-Aad G, Chi Y-L, Raleigh V, Klazinga N. OECD Reviews of Health Care Quality: Israel; Executive Summary, Assessment and Recommendations. 14 October 2012. 30 pp. Available at: http://www.oecd.org/israel/ReviewofHealthCareQualityISRAEL_ExecutiveSummary.pdf
[25] Note: This also was mentioned in Section 3, Study Programs.

competencies before being able to practice fully independently. Alternatively, if one keeps time as a constant, then shifting to a competence-based curriculum with defined outcomes should allow learners on average at any given point in time to have gained and demonstrated greater mastery than now occurs in important competencies. A pilot of this approach is also occurring in a U.S. medical school.

Recommendations:

- Israel should develop a national plan or framework for the composition of its health care workforce and review and revise it at regular intervals. With respect to the physician component of the health care workforce, it will be necessary to anticipate the country's needs for different types of physicians. All government ministries involved in the education of the workforce and its subsequent employment should be involved in the planning.[26]
    - As a corollary to this recommendation, there must be an explicit and thorough examination of the overall trajectory for educating a physician. It is likely that such an examination, and regular reviews, will lead to more effective and efficient physician education process. It also is almost certain to require changing the methods of teaching and assessing medical students and physicians still in training.

- Israel specifically should reconsider the funding model for medical education – not only at the undergraduate level but including all of those involved in the education of physicians. This is a very complex but important task.[27] Failure to do this could lead to not educating effectively or efficiently the physician workforce needed for the 21st century.

---

[26] The authors of the 2010 Carnegie Foundation report on medical education reform made a similar recommendation for the U.S., namely that "AAMC, AMA, ACGME, medical specialty societies, and medical schools collaborate on the development of a medical workforce policy for the United States. A variety of interventions addressing the cost of medical education, length of training, and practice viability ensure that the country has the mix of specialty and subspecialty physicians to meet the needs of the population."

[27] Again, the authors of the 2010 Carnegie Foundation report on medical education reform specifically recommended, "Deans of medical schools and CEOs of teaching hospitals collaboratively make funding for medical education transparent, fair and aligned with the missions of both medical schools and teaching hospitals." It is outside the Committee's scope to suggest the various ways in which the funding model could be changed. There are many possible models. For example, Israel could fund different components of medical education such as preclinical education, clinical education, and research through different funding streams or mixtures of funding streams, e.g., some through universities and some through the health care system. This approach alone has many facets. The current notion of three years of preclinical and three years of clinical education could be re-thought and century. Conversely, doing it well could lead to a reconceptualization not just of funding but of the ways to best educate the needed physician workforce, the integration of science and clinical knowledge and skills, the venues for medical education (ambulatory vs. hospital settings), and the generation of cutting-edge new knowledge.changed. In a mixed model, providers of health care services might need to understand that a portion of the funding they receive is earmarked to support teaching, or to support research by their clinicians, or to support various "academic benefits" such as sabbaticals. The main consideration is that in the end all parties understand what is being funded, by whom, and to what particular end.

Chapter4:   Summary of Recommendations

The Committee lists here the recommendations from each of the sections in Chapter 3. It has not assigned a specific timeline to each. It does feel, however, that action on each recommendation should begin immediately. An important first step would be a decision to go forward on each recommendation and for those on which action will be taken to develop as short a timeline as possible.

Section 1 – Mission

- Consistently reflect each school's overall goals in both the school's mission statement and within the content of its study program.

- A commitment to fostering professionalism among its administration, faculty and students should be stated in the mission of each medical school and reflected throughout the School's activities. (also see Section 4, Faculty)

Section 2 – Organizational Structure

- CHE should review each of the Committee's individual reports in relation to section 3 of its own Standards for Evaluation of Medical Schools and Medical Education.

- CHE needs to review and define clearly the roles of the Deans of Medical Schools, taking into account not only their responsibilities for research and education tasks, a role common to that of deans of other Faculties, but also their professional training roles in medicine and other professions if the Faculty encompasses more than one profession. Given the uncertainty about the capability of an electoral process and short timescale to deliver suitable candidates, it may be that an appointment process or an extended term of elected office may serve better than the present system to ensure that all medical schools have appropriate chief academic officers.

- Require each university that has a Faculty of Medicine or Health Sciences encompassing students in different health professions to submit a report on how its governance effectively represents the needs of each and all of its types of students, how it is or is planning to develop interprofessional education (IPE), and how it already has, or plans to have, resources that can be used by multiple schools to enhance the effectiveness and efficiency of their educational programs.

Section 3 – Study Programs

- CHE should charge a work group with formally assessing the degree to which the English-language medical school study programs compete for scarce resources that are needed for meeting national needs. The scope of the work group should also include assessment of the revenue vs. costs of the English-language programs and the degree to which funding to the universities and their medical schools would need to increase should these programs no longer exist.

- Review/assess the educational effectiveness of the English language programs as compared with the Hebrew programs, particularly for clinical education where language barriers could be significant.

- There should be a formal assessment of the seventh year (stage) as part of the undergraduate education of a physician.

- To enhance the effectiveness and efficiency of medical education there should be a work group that formally assesses the overall trajectory of educating a physician in Israel and analyzes alternatives to the current one. This is likely to require examination of alternative teaching methods and teaching sites. It should be accompanied by assessments of both educational outcomes and costs.

- A systems approach to the early years of medical education, the "preclinical years", should be encouraged in all medical schools as should collaboration in that teaching by basic science and clinical faculty members.

- The 2007 recommendation that "CHE or another national agency play a major role in designing and implementing the collaboration between medical education and health-care providers" should be broadened so that it is not just about ambulatory health-care teaching but about all the resources needed for effective medical and other health professions education. The current Committee is not aware that the 2007 recommendation was implemented across all schools and urges that it be implemented now.

Section 4 – Human Resources/Faculty

- Every school should have a policy that every physician who is hired to teach medical students and residents should have already demonstrated excellent professional values, understand that part of the job is to infuse professional values in all contacts with learners, and be aware that demonstrated professionalism is a requirement for eligibility for promotion.

- CHE should encourage each medical school to have a promotions process that explicitly considers the quality of teaching performance for any faculty

member who is expected to teach. Implementing this recommendation will require each school to develop suitable, auditable assessments of teaching and professional performance. Furthermore, even those faculty members whose promotion is under consideration primarily because of research excellence should have demonstrable skills in teaching that are satisfactory or better.

- CHE should encourage each university to develop a promotions process for its Faculty of Medicine or Health Sciences that values the multiple types of academic excellence and scholarship necessary for effective medical and health professions education. In addition to having a track for promotion that values traditional forms of investigation, there should be tracks that value teaching and educational scholarship, and clinical innovation including programmatic innovation.

- CHE should encourage each Faculty of Medicine or Health Sciences to create a portfolio of faculty development and remediation activities and demonstrate that faculty performance is improving in all domains of medical education. Faculty development should also be facilitated by the new national organization of medical educators and having it be open to all interested in medical education practices and research. There needs to be sustainable support for such an organization. (see Section 6, Teaching and Learning Outcomes)

Section 5 – Students

- CHE should have a work group evaluate the degree to which students in Israeli medical schools need to take jobs for financial reasons, and if there is significant financial burden consider policies that would alleviate that burden. The work group could also consider the ways in which jobs for medical students could best be structured to complement their medical education.

- Committee recommendations regarding teaching and assessment methods that enhance student learning can be found in Section 6, Teaching and Learning Outcomes.

Section 6 – Teaching and Learning Outcomes

- All schools must articulate appropriate overall learning outcomes (competencies) for their curriculum, and should utilize them as the basis for individual course and clerkship objectives and for assessments, with all

learning activities and expected outcomes explicitly linked to specific learning objectives. This process might be facilitated through collaboration at the national level.

- Reduce dependence on frontal lectures, providing more opportunities for interactive learner-centered small group and discussion formats (problem-based and team-based learning methods, for example) and for learning in clinical settings, even early in the curriculum.

- Provide sufficient and appropriate opportunities for clinical education in ambulatory settings, and especially in primary care.

- Provide students with opportunities for more active involvement in patient care activities, in both inpatient and ambulatory settings.

- Reduce dependence on multiple choice examination formats for student assessment. Expand the use of performance-based assessments, using faculty observation, patient instructors, or other clinical simulation methods as appropriate. Wherever individual students or small groups have contact with a faculty member over a period of a few weeks or more, the student should receive formal, written performance feedback.
    - National examinations in clinical fields already exist but have only a multiple choice format. Each national examination should utilize additional assessment methods, e.g., an OSCE, to determine that the desired threshold of clinical competence has been achieved or exceeded by students.

      Consideration also should be given to developing ways of assessing that students in all study programs, including the English-language programs, have achieved or exceeded the desired thresholds of competence.

- Each of the schools is underutilizing clinical simulation methods for teaching as well as performance assessment. Further resource development, from small task-trainers to whole-body simulators, and including patient-instructor/standardized patient methods, is needed. This is another area in which a nationwide collaboration, utilizing resources such as are already available at the MSR clinical simulation center, may be helpful.

- Work toward meaningful integration of the curriculum, both horizontal and vertical. Ensure appropriate central oversight and authority for curricular change. Consider changes to the baccalaureate degree requirements so as to allow the introduction of clinical perspectives and skills in the early curricular years.

- Rebuild and invest in medical education centers to provide needed expertise in faculty development, curricular and instructional design, and assessment.

- Encourage collaboration at the national level in carrying out several of these recommendations, particularly in the areas of curricular development and performance assessment.

- Nurture the new organization dedicated to scholarship in medical education, support and sustain it, so that it can help in developing a national infrastructure for medical education.

Section 7 – Research

- MD-PhD programs should only be authorized if they provide the expectation that the time allotted and supported for the PhD is a minimum of three years.

- The Dean's Forum in which all five Faculty of Medicine/Health Sciences deans participate should compare the MD-PhD programs and consider best practices and possible ways to attract a substantial and stable group of students to those programs to the degree that they meet a national need for physician researchers.

- The Dean's Forum should consider developing a uniform policy that can be recommended for adoption to each parent institution that would encourage persons who obtain MD-PhDs or PhDs in medical sciences at that institution to obtain appropriate postdoctoral training and experience.

- The Dean's Forum should compare the undergraduate thesis programs across the schools and attempt to assess their educational value for all medical students and find and spread best practices.

Section 8 – Infrastructure

- CHE should foster consideration of how existing national resources such as MSR could assist medical schools throughout the country in expanding their use of simulation in medical education.

- CHE should foster consideration of how existing local resources such as the multi-media program at HUJI could be helpful in medical education programs elsewhere in the country and how best to transform a local resource into a national one.

Section 9 – Evaluation (National and Local)

- The Committee strongly recommends that CHE adopt processes consistent with the WFME recognition guidelines for accreditation authorities, and do so in advance of the date (2023) when ECFMG will require such recognition. This will no doubt alter the expectations Israeli schools must meet for subsequent quality assurance review cycles. The definition of new accreditation standards and timetable for their implementation would follow once new overall processes are in place.

    In approaching this challenge, we suggest that CHE convene a committee representing appropriate stakeholders such as Israeli medical professional organizations, medical education leaders, and governmental authorities, and also some international experts. The purposes would be to review current CHE policies and procedures in light of international expectations for accreditation of medical training and create a proposal to address these issues.

- WFME is including a parallel process related to postgraduate medical training and similar issues are applicable to other health professions overseen by the CHE. We recommend that CHE familiarize itself with these and address them as well.

Section 10 – Additional Important Policy Considerations

- Israel should develop a national plan or framework for the composition of its health care workforce and periodically revise it. With respect to the physician component of the health care workforce, it will be necessary to anticipate the country's needs for different types of physicians. All government ministries involved in the education of the workforce and its subsequent employment should be involved in the planning.
    - As a corollary to this recommendation, there must be an explicit and thorough examination of the overall trajectory for educating a physician. It is likely that such an examination, and regular reviews, will lead to more effective and efficient physician education process. It also is almost certain to require changing the methods of teaching and assessing medical students and physicians still in training.

- Israel specifically should reconsider the funding model for medical education – not only at the undergraduate level but including all of those involved in the education of physicians. This is a very complex but important task. Failure to do this could lead to not educating effectively or efficiently the physician workforce needed for the 21st century.

Signed by:

Prof. Stephen Schoenbaum – Chair

Prof. Peter Crome

Prof. Raymond H Curry

Prof. Elliot Gershon

Prof. Shimon Glick

Prof. David Katz

Prof. Ora Paltiel – Clarfield

Prof. Jo Shapiro

Appendix 1: Letter of Appointment

February 2014

Prof. Stephen Schoenbaum
The Josiah Macy Jr. Foundation,
USA

Dear Professor Schoenbaum,

The Israeli Council for Higher Education (CHE) strives to ensure the continuing excellence and quality of Israeli higher education through a systematic evaluation process. By engaging upon this mission, the CHE seeks: to enhance and ensure the quality of academic studies, to provide the public with information regarding the quality of study programs in institutions of higher education throughout Israel, and to ensure the continued integration of the Israeli system of higher education in the international academic arena.

As part of this important endeavor we reach out to world renowned academicians to help us meet the challenges that confront the Israeli higher education by accepting our invitation to participate in our international evaluation committees. This process establishes a structure for an ongoing consultative process around the globe on common academic dilemmas and prospects.

I therefore deeply appreciate your willingness to join us in this crucial enterprise.

It is with great pleasure that I hereby appoint you to serve as the Chair of the Council for Higher Education's Committee for the Evaluation of the study programs in **Medical Studies**. In addition to yourself, the composition of the Committee will be as follows: Prof. Peter Crome, Prof. Raymond Curry, Prof. Shimon Glick, Prof. Jo Shapiro, Prof. Eliot Gershon, Prof. David Katz and Prof. Ora Paltiel-Clarfield.

Ms. Daniella Sandler will be the coordinator of the committee.

Details regarding the operation of the committee and its mandate are provided in the enclosed appendix.

I wish you much success in your role as the Chair of this most important committee.

Sincerely,

Prof. Hagit Messer-Yaron
Deputy Chairperson,
The Council for Higher Education (CHE)

Enclosures: Appendix to the Appointment Letter of Evaluation Committees

cc: Ms. Michal Neumann, Deputy Director-General for QA, CHE
Ms. Daniella Sandler, Committee Coordinator

Appendix 2: Site Visit Dates

Ben-Gurion University	February 24 – 26, 2014
The Technion	March 2 – 4, 2014
Tel Aviv University	May 19 – 21, 2014
Hebrew University – Hadassah	May 25 – 27, 2014
Bar-Ilan University	June 1 – 2, 2014

Appendix 3:

Standards for Evaluation of Medical Schools and Medical Education

1. Objectives

The main objective of a program of medical education leading to the M.D. degree in Israel is to prepare its graduates to enter and complete graduate medical education, to qualify for licensure, to provide competent medical care, and to have the educational background necessary for continued learning. A medical school may establish additional objectives for its educational program, consistent with its program resources. A medical school must define its objectives and make them known to faculty and students.

2. Governance

A medical school should be a component of a university that has other graduate and other professional degree programs. The program of medical education leading to the M.D. degree must be conducted in an environment that fosters the intellectual challenge and spirit of inquiry as characterized by the community of scholars that constitutes a university.

A medical school in Israel must be part of a non-profit university.

3. Administration

The administration of a medical school must be effective and appropriate in light of the main objectives and its particular mission and objectives.

The chief academic official of a medical school must be qualified by education and experience to provide leadership in medical education.

The faculty of a medical school must be appropriately qualified to teach and be involved in decisions involving admissions and curriculum.

Administrative officers and members of a medical school faculty are appointed by, or on the authority of, the governing board of the medical school or its parent university.

The chief official of the medical school, who usually holds the title "dean," must have ready access to the university president or other university official charged with final responsibility for the school, and to other university officials as are necessary to fulfill the responsibilities of the dean's office.

The dean must be qualified by education and experience to provide leadership in medical education, in scholarly activity and research, and in care of patients. The dean should have the assistance of such associate or assistant deans and staff as are necessary for administration of admissions, student affairs, academic affairs, graduate education, continuing education, hospital relationships, research, business and planning, and fund raising.

The manner in which the medical school is organized, including the responsibilities and privileges of administrative officers, faculty, students, and committees must be promulgated in medical school or university bylaws. A committee structure is the usual mechanism for involving faculty and others in decisions concerning admissions, promotions, curriculum, library and research, etc. The names, membership, and functions of such committees are not prescribed by these standards, but rather are subject to local determination and needs.

In determining the appropriate organization, emphasis should be placed on the importance of the collegiality of the medical school faculty responsible for undergraduate medical education and for the continuum of medical education. Ambulatory consideration should be given to the commitments of faculty members who have multiple academic responsibilities in several educational programs of a complex university, so as to assure each educational program adequate faculty resources. A decision must be made concerning the provision of a single faculty or of combined faculties to serve the needs of each of several health-related or other academic programs of the university, and concerning the advisability of joint faculty appointments. The school must ensure that appointments such as “clinical professors”, must be made according to approved academic criteria.

4. Geographically Separated Programs

If components of the program are conducted at sites geographically separated from the main campus of the medical school, the chief academic officer of the medical school must be responsible for the conduct and maintenance of the quality of the educational experience offered at these sites and for identification of the faculty at all sites. The principal academic officer of each geographically separated site must be administratively responsible to the chief academic officer of the medical school conducting the accredited program. The faculty in each discipline, in all sites, must be functionally integrated by administrative mechanisms that ensure comparable quality in the educational experiences

and consistency in student evaluation at the geographically separated segments of the program.

A large number of program sites or a significant distance between sites may require extra academic and administrative controls in order to maintain the quality of the entire program.

A medical school must specify its separated campuses and the faculty teachers in them.

5. Educational program for the M.D. degree

The educational program of a medical school must be of sufficient length to meet the main objectives and its particular mission and objectives, and to provide students with knowledge and skills necessary to become qualified physicians.

The curriculum must provide a broad-base education in the science basic to medicine, a variety of clinical subjects, and various ethical, behavioral and socioeconomic subjects pertinent to medicine.

The requirements for successful completion of the program of medical education must include a particular focus on clerkships and other form of clinical training. Students must have hands-on experience.

5.1. Duration

The program of education in the art and science of medicine leading to the M.D. degree must be of 6 years duration plus one year of rotating internship. Additionally, The CHE approved that institutions will be able to submit a proposal to establish a four-year study program in Medicine (with an additional internship year) for the "Medical Doctor" (M.D.) degree, designated for students that hold of the "Bachelor of Science" degree.

5.2. Design and Management

The program's faculty is responsible for the design, implementation, and evaluation of the curriculum. There must be integrated institutional responsibility for the design and management of a coherent and coordinated curriculum. The chief academic officer must have sufficient available resources and authority provided by the institution to fulfill this responsibility. The curriculum of the program leading to the M.D. degree must be designed to provide a general professional education, recognizing that this alone is insufficient to prepare a graduate for independent, unsupervised practice. Medical schools must evaluate educational program effectiveness by documenting the achievement of their students and graduates in verifiable and internally consistent ways that show the extent to which institutional and program purposes are met.

The committee responsible for curriculum should give careful attention to the impact on students of the amount of work required. The committee should monitor the content provided in each discipline in order that objectives for education of a physician are

achieved without attempting to present the complete, detailed, systematic body of knowledge in that discipline. The objectives, content, and methods of pedagogy utilized for each segment of the curriculum, as well as for the entire curriculum, should be subjected to periodic evaluation. Redundancies and deficiencies in the curriculum identified by the evaluations should be corrected.

Medical schools should use a variety of measures to evaluate program quality, such as data on student performance, academic progress and program completion rates, acceptance into residency programs, postgraduate performance, and emerging measures that may prove to be valid. The results of such evaluations should be used to determine how well schools are fulfilling their objectives and to assess the need for program improvement. Schools also should evaluate the performance of their students and graduates in the framework of national norms of accomplishment. Review and necessary revision of the curriculum is an ongoing faculty responsibility.

5.3. Content

The medical faculty is responsible for devising a curriculum that enables students to learn the fundamental principles of medicine, to acquire skills of critical judgment based on evidence and experience, and to develop an ability to use principles and skills wisely in solving problems of health and disease. In addition, the curriculum must be designed so that students acquire an understanding of the scientific concepts underlying medicine. In designing the curriculum, the faculty must introduce current advances in the basic and clinical sciences, including therapy and technology, changes in the understanding of disease, and the effect of social needs and demands on medical care.

The curriculum cannot be all-encompassing. However, it must include the sciences basic to medicine, a variety of clinical disciplines, and ethical, behavioral, and socioeconomic subjects pertinent to medicine. There should be presentation of material on medical ethics and human values. The faculty should foster in students the ability to learn through self-directed, independent study throughout their professional lives.

The curriculum must include the contemporary content of those expanded disciplines that have been traditionally titled anatomy, biochemistry, physiology, microbiology and immunology, pathology, pharmacology and therapeutics, and preventive medicine. Instruction within these basic sciences should include laboratory or other practical exercises which facilitate the ability to make accurate quantitative observations of biomedical phenomena and critical analyses of data. Teachers and teaching assistants in the biomedical sciences must be familiar with the educational objectives of the course and be prepared for their roles in teaching and evaluation.

All schools must provide broad-based clinical education programs that equip students with the knowledge, skills, attitudes, and behaviors necessary for further training in the practice of medicine. Instruction and experience in patient care must be provided in both ambulatory and hospital settings. All schools must offer a core curriculum in primary care, utilizing the disciplines or multidisciplinary approaches involved in the delivery of such care.

Clinical education programs should include disciplines such as family medicine, internal medicine, obstetrics and gynecology, pediatrics, psychiatry, and surgery. Schools must ensure that their students possess the knowledge and clinical abilities to enter any field of graduate medical education. Clinical instruction should cover all organ systems, and must include the important aspects of acute, chronic, continuing, preventive, and rehabilitative care.

The faculty must participate in a process that defines the objectives of clinical education and establishes quantified criteria for the types of patients (real or simulated), the level of student responsibility, and the appropriate clinical settings necessary to accomplish these purposes. A system for monitoring the achievement of clinical educational goals must be developed, based on these criteria, and students must be evaluated in this framework. If the level or diversity of student interactions with patients does not meet the school-based criteria, specific mechanisms must be in place to adjust the criteria or to alter the educational program. Either may be done only within appropriate, documented means that ensure continued educational quality.

The curriculum must provide grounding in the body of knowledge represented in the disciplines that support the fundamental clinical subjects, for example, diagnostic imaging and clinical pathology. Students must have opportunities to gain knowledge in those content areas that incorporate several disciplines in providing medical care, for example, emergency medicine and the care of the elderly and disabled. In addition, students should have the opportunity to participate in research and other scholarly activities of the faculty.

Each required clinical clerkship must allow the student to undertake a thorough study of a series of selected patients having the major and common types of disease problems represented in the primary and related disciplines of the clerkship. The committee responsible for curriculum must require close faculty supervision of the learning experience of each student at the appropriate level of graded clinical responsibility. Supervision must be provided throughout required clerkships by members of the school s faculty. The required clerkships should be conducted in a teaching hospital or ambulatory care facility where residents in accredited programs of graduate medical education, under faculty guidance, may participate in teaching the students. Residents must be fully informed about the educational objectives of the clerkships and be prepared for their roles as teachers and evaluators of medical students. In an ambulatory care setting, if faculty supervision is present, resident participation may not be required. If required clerkships in a single discipline are conducted in several hospitals, every effort must be made to ensure that the students receive equivalent educational experiences.

The faculty committee responsible for curriculum should develop, and the chief academic officer should enforce, the same rigorous standards for the content of each year of the program leading to the M.D. degree. The final year should complement and supplement the curriculum so that each student will acquire appropriate competence in general medical care regardless of subsequent career specialty. The curriculum should include

elective courses designed to supplement the required courses and to provide opportunities for students to pursue individual academic interests. Faculty advisors must guide students in the choice of elective courses. If students are permitted to take electives at other institutions, there should be a system centralized in the dean s office to screen the students' proposed extramural programs prior to approval and to ensure the return of a performance appraisal by the host program. Another system, devised and implemented by the dean, should verify the credentials of students from other schools wishing to take courses or clerkships at the school, approve assignments, maintain a complete roster of visiting students, and provide evaluations to the parent schools.

All instruction should stress the need for students to be concerned with the total medical needs of their patients and the effect on their health of social and cultural circumstances.

The school must specify how students are prepared for their role in addressing the medical consequences of common societal problems, for example, providing instruction in the diagnosis, prevention, appropriate reporting and treatment of violence and abuse. Students must be encouraged to develop and employ scrupulous ethical principles in caring for patients, in relating to patients families, and to others involved in the care of the patients. These principles are essential if the physician is to gain and maintain the trust and respect of patients, colleagues, and the community.

In view of the increasing pace of discovery of new knowledge and technology in medicine, The Council for Higher Education encourages experimentation that will increase the efficiency and effectiveness of medical education. Experiments should have carefully defined goals and plans for implementation, including methods of evaluating the results. Planning for educational innovation should consider the incremental resources that will be required, including demands on library facilities and operation, information management needs and computer hardware and software.

5.4. Evaluation of Student Achievement; Due Process

The medical school faculty must establish principles and methods for the evaluation of student achievement, and make decisions regarding promotion and graduation. The evaluation of student achievement must employ a variety of measures of knowledge, competence and performance, systematically and sequentially applied throughout medical school. Each provisionally accredited program must utilize methods for determining the quality of its program and the level of achievement of its students compared to national norms.

The chairman of each discipline should set the standards of achievement by students in the study of that discipline. Narrative descriptions of student performance and of non-cognitive achievements should be recorded to supplement grade reports in all required clinical clerkships and in all courses where student-faculty interaction permits this form of assessment. The faculty committee should review the frequency of examinations and their scheduling, particularly when the students are enrolled in several subjects simultaneously. The Council for Higher education urges schools to develop a system of

evaluation that fosters self-initiated learning by students, and disapproves of the use of frequent tests which condition students to memorize details for short-term retention only. Examinations (written and others) should measure cognitive learning, mastery of basic clinical skills, and the ability to use data in realistic problem solving. Institutions must develop a system of assessment which assures that students have acquired and can demonstrate on direct observation the core clinical skills and behaviors needed in subsequent medical training. Communication skills are integral to the education and effective function of physicians. There must be specific instruction and evaluation of these skills as they relate to physician responsibilities, including communication with patients, families, colleagues and other health professionals.

There must be comparable educational experiences and equivalent methods of evaluation across all alternative instructional sites within a given discipline. If geographically separated campuses are operated, a single standard for promotion and graduation of students should be applied.

The medical school must publicize to all faculty members and students its standards and procedures for the evaluation, advancement, and graduation of its students and for disciplinary action. There should be a fair and relatively formal process for the faculty or administration to follow when taking any action that adversely affects the status of a student. The process should include timely notice of the impending action, disclosure of the evidence on which the action would be based, and an opportunity for the student to respond. A student's records must be available for review by the student, and the student must have the right and be given the opportunity to challenge the accuracy of the record. Student records must be confidential and should be made available only to members of the faculty and administration with a need to know, unless released by the student, or as otherwise governed by laws concerning confidentiality.

5.5. Academic Counseling and Career Guidance

The chief academic officer and the directors of all courses and clerkships must design and implement a system of evaluation of the work of each student during progression through each course or clerkship. Each student should be evaluated early enough during a unit of study to allow time for remediation. Course directors and faculty assigned to advise students should consider this duty a primary responsibility. All course directors or departmental heads, or their designates, should serve as expert consultants to the chief academic officer for facilitation of performance of both students and faculty.

The Council will evaluate the programs designs to assist students in selecting a future medical career and in developing a strategy for application to residency programs. Any such system should not disrupt a student's curriculum in general medical education by external pressures to make premature application to residency programs.

6. Medical students

Medical school must admit only those students who possess the intelligence, integrity, and personal characteristics that are generally perceived as necessary to become effective physicians.

Medical school must carefully monitor the progress of students through the educational program and graduate only those students who successfully complete the program.

6.1. Admissions

The faculty of each school should develop criteria and procedures for the selection of students, which should be published and available to potential applicants. To further the accomplishment of its purposes, each medical school should have policies and practices addressing the gender, racial, cultural, and economic diversity of its students. Medical schools must strive to select students who possess the intelligence, integrity, and personal and emotional characteristics that are perceived necessary for them to become effective physicians.

While physical disability should not preclude a student from consideration for admission, each school should develop and publish technical standards for the admission of handicapped applicants, in accordance with legal requirements.

The selection of students for the study of medicine is the responsibility of the medical school faculty through a duly constituted committee. Persons or groups external to the medical school may assist in the evaluation of applicants, but the final responsibility must not be delegated outside the medical faculty. There must not be any political or financial influence on the selection of students. All factors utilized in the selection process must be made public.

A medical school's publications, advertising, and student recruitment should present a balanced and accurate representation of the mission and objectives of the educational program. The catalog or equivalent informational materials must describe all courses offered by the school, a complete description of the requirements for the M.D. degree and all associated degrees, the most recent academic calendar for each of the curricular options available, a description of the admissions process, and the enumeration of criteria used in the selection of students.

There must be no discrimination on the basis of sex, age, race, creed or national origin. Compliance with both written and implied public policy must be assured. The student body should be drawn from a wide spectrum of economic backgrounds. Advanced standing may be granted to students for work done prior to admission.

Each medical school or its parent university should define the standards of conduct in the teacher-learner relationship. Schools should develop and widely promulgate written procedures that allow medical students to report violations of these standards--such as incidents of harassment or abuse--without fear of retaliation. The procedures also should specify mechanisms for the prompt handling of such complaints, and for the educational methods aimed at preventing student mistreatment.

6.2. Geographically separated campus

If geographically separated campuses are operated, the selection and assignment of all medical students is the ultimate responsibility of the degree granting school. Within reasonable limits, students should have the opportunity to move between the component programs of the school.

Recognizing that quality and quantity of educational opportunities may vary between components, it is recommended that transfer students with advanced standing be assigned for at least half their first academic year to that component of the school which offers the most complete program and broadest variety of resources and experiences.

Students assigned to a branch campus should receive the same privileges and access to student services as students on the main campus.

6.3. Transfer and visiting students

Differences in curricula across schools require that decisions about the transfer of students between schools be based on an assurance that the courses previously taken are compatible with the program to be entered. Accepted transfer students must have demonstrated achievements in premedical education and medical school that are comparable to those of students in the class they join. There must be sufficient institutional resources to accommodate the transfer of students. Transfer students may be accepted into the middle years of the curriculum, but must not be accepted into the final year of the program except under rare circumstances.

Students visiting from other schools for clinical clerkships and electives must possess qualifications equivalent to the students they will join in these experiences. There must be sufficient institutional resources to accommodate such students without significantly diminishing the resources available to students already enrolled. Visiting students must be registered by the school for the period in which they are visiting, so as to ensure that they satisfy the school's requirements for health records, immunizations, health insurance, and liability protection.

6.4. Financial Aid; Tuition and Fee Refund Policy

A school must report its policies with regard to counseling about financial aid. To the extent possible, a school should provide financial aid to students.

6.5. Amenities for Students

A school should provide students with amenities that increase efficiency, such as study space, lounge areas and food service, if not available in the immediate vicinity of the school. The medical school should have an appropriate security system for its personnel and all properties.

6.6. Personal Counseling; Student Health Services

A school must report its system of personal counseling for students. The faculty and administrators should determine whether personal counsel is to be provided by an officer of administration, by assignment of faculty members or others for this purpose, or both.

There must be a system for preventive and therapeutic health services to students, to make health insurance available to all students and their dependents, and to make

disability insurance available to students. Schools must develop policies dealing with students' exposure to infectious and environmental hazards. The policies must include: (1) education of students about methods of prevention; (2) the procedures for care and treatment after exposure, including definition of financial responsibility; (3) and the effects of infectious and/or environmental disease or disability on student education activities. Confidential counseling by mental health professionals must be available to students.

7. Resources for the educational program

Medical school must have physical facilities that are qualitatively adequate for the size and scope of the educational program, as well as of the student body.

The faculty must provide effective teaching and be of sufficient size to provide the scope of the educational program offered.

Medical school must have a library sufficient in size, breadth and depth to support the educational program.

7.1. General Facilities

A medical school must have, or be assured use of, buildings and equipment that are quantitatively and qualitatively adequate to provide an environment conducive to high productivity of faculty and students. Geographic separation between facilities may be dysfunctional. The facilities must include faculty offices and research laboratories, student classrooms and laboratories, amenities for students, offices for administrative and support staff, and a library. Access to an auditorium sufficiently large to accommodate the student body is desirable. The school should be equipped to conduct biomedical research and must provide facilities for humane care of animals when animals are used in teaching and research.

7.2. Faculty

Members of the faculty must have the capability and continued commitment to be effective teachers. Effective teaching requires knowledge of the discipline and an understanding of pedagogy, including construction of a curriculum consistent with learning objectives, subject to internal and external formal evaluation. The administration and the faculty should have knowledge of methods for measurement of student performance in accordance with stated educational objectives and national norms.

Persons appointed to a faculty position must have demonstrated achievements within their disciplines commensurate with their faculty rank. The recruitment and development of a medical school's faculty should take into account its mission, the diversity of its student body, and the populations that it serves. It is expected that faculty members will have a commitment to continuing scholarly productivity, thereby contributing to the educational environment of the medical school.

In each of the major disciplines basic to medicine and in the clinical sciences, a critical mass of faculty members must be appointed who possess, in addition to a comprehensive knowledge of their major disciplines, expertise in one or more subdivisions or specialties within each of these disciplines. In the clinical sciences, the number and kind of specialists appointed should relate to the amount of patient care activities required to conduct meaningful clinical teaching at the undergraduate level, as well as for graduate and continuing medical education.

Physicians practicing in the community can make a significant contribution to the educational program of the medical school, subject to individual expertise, commitment to medical education, and availability. Practicing physicians appointed to the faculty, either on a part-time basis or as volunteers, should be effective teachers, serve as role models for students, and provide insight into contemporary methods of providing patient care. The quality of an educational program is enhanced by the participation of volunteer faculty in faculty governance, especially in defining educational goals and objectives.

There must be clear policies for the appointment, renewal of appointment, promotion, granting of tenure and dismissal of members of the faculty. The appointment process must involve the faculty, the appropriate departmental heads, and the dean. Each appointee should receive a clear definition of the terms of appointment, responsibilities, line of communication, privileges and benefits, and policy on practice earnings. Faculty members should receive regularly scheduled feedback on their academic performance and their progress towards promotion. Opportunities for professional development should be provided to enhance faculty members skills and leadership abilities in teaching and research.

The education of both medical students and graduate physicians requires an academic environment that provides close interaction between faculty members, so that those skilled in teaching and research in the basic sciences can maintain awareness of the relevance of their disciplines to clinical problems. Such an environment is equally important for clinicians, for from the sciences basic to medicine comes new knowledge which can be applied to clinical problems. A medical school should endeavor to provide a setting in which all faculty members work closely together in teaching, research, and health care delivery, to disseminate existing knowledge and to generate new knowledge of importance to the health and welfare of mankind.

Graduate medical education and graduate education in the biomedical and behavioral sciences are important parts of the academic environment of a medical school. There should be regular institutional review of the graduate programs in which medical school faculty participate, addressing the quality of education, the research and scholarship of the faculty, and the progress and achievement of the trainees.

The dean and a committee of the faculty should determine medical school policies. This committee typically consists of the heads of major departments, but may be organized in any manner that brings reasonable and appropriate faculty influence into the governance and policymaking processes of the school. The full faculty should meet often enough to

provide an opportunity for all to discuss, establish, and otherwise become acquainted with medical school policies and practices.

A medical school should have policies which deal with circumstances in which the private interests of its faculty or staff may conflict with their official responsibilities.

7.3. Library

The medical school must have a well-maintained and catalogued library, sufficient in size and breadth to support the educational programs offered by the institution. The library should receive the leading biomedical and clinical periodicals, the current numbers of which should be readily accessible. The library and any other learning resources should be equipped to allow students to learn new methods of retrieving and managing information, as well as to use self-instructional materials. A professional library staff should supervise the library and provide instruction in its use.

If the library serving the medical school is part of a medical center or university library system, the professional library staff must be responsive to the needs of the medical school, its teaching hospitals, the faculty, resident staff, and students who may require extended access to the journal and reference book collections. The librarian should be familiar with the methods for maintaining relationships between the library and national library systems and resources, and with the current technology available to provide services in non-print materials. If the faculty and students served by the library are dispersed, the utilization of departmental and branch libraries should be facilitated by the librarian and by the administration and faculty of the school.

The library should also be a community resource in support of continuing medical education.

7.4. Clinical Teaching Facilities

The medical school must have adequate resources to provide clinical instruction to its medical students. Resources must include ambulatory care facilities and hospitals where the full spectrum of medical care is provided and can be demonstrated. Each major clinical department must have a residency program accredited by the Israel Medical Association Scientific Council. The number of hospital beds required for education cannot be specified by formula, but the aggregation of clinical resources must be sufficient to permit students in each of the major clerkships to work up and follow several new patients each week.

Since undergraduate medical education usually requires the conduct of simultaneous and mutually supportive programs of graduate medical education, clinical facilities must be adequate for both parts of the continuum of medical education. A hospital that provides a base for the education of both medical students and residents must have adequate library resources, not only for the clinical staff, but also for the faculty and the students. Ready access to areas for individual study, for conferences, and for lectures is necessary.

The nature of the relationship of the medical school to affiliated hospitals and other clinical resources is extremely important. There should be written affiliation agreements that define the responsibilities of each party. The degree of the school's authority should reflect the extent that the affiliated clinical facility participates in the educational programs of the school. Most critical are the clinical facilities where required clinical clerkships are conducted. In affiliated institutions, the school's department heads and senior clinical faculty members must have authority consistent with their responsibility for the instruction of students.

Recognizing the special relationship between the medical school and its affiliated teaching hospitals, it is imperative that the academic programs remain under the control of the faculty in all medical school-hospital relationships.

****
